# Integrated Molecular Diversity and Aroma-Related Volatile Compound Profiling of Thai Rice Cultivars (*Oryza sativa* L.) Using ISSR, SCoT and SRAP Markers

**DOI:** 10.3390/plants15213482

**Published:** 2026-11-07

**Authors:** Juthaporn Saengprajak, Pimradawan Kulchotchaiwong, Aphidech Sangdee

**Affiliations:** Department of Biology, Faculty of Science, Mahasarakham University, Kantarawichai, Maha Sarakham 44150, Thailand; 65010217009@msu.ac.th (P.K.); aphidech.s@msu.ac.th (A.S.)

**Keywords:** DNA fingerprinting, germplasm characterization, HS-GC-MS, *Oryza sativa* L., tice aroma, volatile compounds

## Abstract

Aromatic rice is highly valued worldwide for its distinctive fragrance and superior grain quality. Despite the importance of genetic diversity and aroma-related metabolites for germplasm conservation and breeding, studies integrating molecular markers and volatile profiling in Thai rice remain limited. This study integrated ISSR, SCoT, and SRAP molecular markers with static headspace gas chromatography-mass spectrometry (HS-GC-MS) to characterize the genetic diversity and volatile profiles of Thai rice germplasm. Twenty-nine aromatic and non-aromatic cultivars were evaluated using the three molecular marker systems. All marker systems revealed substantial polymorphism, with SRAP showing the highest overall discriminatory performance, including the highest percentage of polymorphic bands (79.19%) and the highest mean PIC, RP, MI, and *H_e_*. The combined marker dataset provided a broader representation of molecular variation, with UPGMA clustering and PCoA differentiating the rice accessions into distinct genetic groups. Four representative cultivars (KDML105, PTT1, RD43, and RD79) were subsequently selected from the 29 rice cultivars for comparative volatile profiling based on their genetic relationships. Six volatile compounds were tentatively identified, comprising four aldehydes, one carboxylic acid, and one ester. Hexanal was the predominant volatile compound across all four cultivars, whereas acetic acid, 4-methylbenzaldehyde, and methyl salicylate differed among the analyzed samples. Multivariate analysis distinguished KDML105 from the other three cultivars based on their volatile profiles. Integrating multilocus DNA fingerprinting with volatile profiling provides complementary insights into the genetic and biochemical diversity of Thai rice germplasm, supporting cultivar characterization, germplasm management, and rice breeding.

## 1. Introduction

Rice (*Oryza sativa* L.) is one of the world’s most important cereal crops and serves as the primary source of dietary energy for more than half of the global population [1,2,3]. In addition to its indispensable contribution to global food security, rice is a major agricultural commodity that plays a vital role in the economies of many Asian countries through both domestic consumption and international trade [2,4]. Increasing consumer demand for premium-quality rice has shifted breeding priorities from maximizing grain yield towards improving grain quality attributes, including cooking quality, texture, nutritional value and aroma. Among these characteristics, grain aroma is regarded as one of the most valuable quality traits because it strongly influences consumer preference, market value and export competitiveness [5,6,7]. Aromatic rice cultivars, particularly Thai jasmine rice (Khao Dawk Mali 105; KDML105) and Basmati rice, are internationally recognized for their distinctive fragrance, superior cooking quality, and premium market value [2,5,8]. Owing to their economic importance and increasing global consumer demand, the conservation, genetic characterization, and effective utilization of aromatic rice germplasm have become major priorities in modern rice breeding programs aimed at developing improved cultivars with enhanced grain quality, stable aroma expression, and broad environmental adaptation [2,8].

Thailand is recognized as one of the world’s major centers of rice diversity, possessing abundant germplasm resources comprising aromatic, non-aromatic, glutinous and colored rice cultivars that are adapted to diverse agroecological environments [8,9,10]. These genetic resources represent valuable reservoirs of alleles associated with grain quality, yield potential, disease resistance and tolerance to biotic and abiotic stresses, providing indispensable resources for crop improvement programs [11]. However, the extensive cultivation of modern high-yielding varieties has accelerated genetic erosion and increased concerns regarding the loss of unique alleles preserved in traditional cultivars and local landraces [12]. Comprehensive characterization of rice germplasm is therefore essential for the conservation of genetic resources, identification of elite parental materials and sustainable utilization of valuable genetic variation in future breeding programs [13].

Traditionally, rice germplasm has been characterized using standardized agro-morphological descriptors, which remain indispensable for cultivar characterization, distinctness, uniformity and stability (DUS) testing, and variety registration. However, the expression of many morphological traits is influenced by environmental conditions, cultivation practices, and genotype × environment interactions, thereby limiting the reliability of morphology alone for discriminating closely related cultivars. Consequently, molecular markers are increasingly employed to complement morphological characterization and improve the accuracy of germplasm identification and genetic diversity assessment [14,15,16,17]. Advances in molecular genetics have enabled the development of DNA-based marker systems that directly detect genetic polymorphisms at the genome level, providing more reliable estimates of genetic diversity than phenotypic traits, which are often influenced by environmental conditions and developmental stages [18,19,20]. Consequently, DNA-based molecular markers have become indispensable tools for cultivar identification, germplasm characterization, genetic diversity assessment, phylogenetic analysis, conservation genetics, and marker-assisted breeding. By directly detecting DNA polymorphisms that are largely independent of environmental influences, these markers provide more accurate and reliable estimates of genetic relationships than morphological traits alone [21,22,23].

Several DNA marker systems have been applied in rice genetic studies, including Restriction Fragment Length Polymorphisms (RFLPs), Amplified Fragment Length Polymorphisms (AFLPs), Random Amplified Polymorphic DNA (RAPD), Simple Sequence Repeats (SSRs), Single-Nucleotide Polymorphisms (SNPs), and PCR-based dominant marker systems [21]. Among these, Inter-Simple Sequence Repeat (ISSR), Sequence-Related Amplified Polymorphism (SRAP), and Start Codon-Targeted (SCoT) markers have gained considerable attention because they are technically simple, reproducible, cost-effective, and capable of detecting polymorphisms across complementary genomic regions [24,25,26,27]. ISSR markers amplify DNA fragments located between adjacent microsatellite loci and are effective for detecting genome-wide polymorphisms, whereas SRAP markers preferentially target open reading frames and other coding regions associated with functional genes. In contrast, SCoT markers amplify conserved sequences flanking the ATG translation initiation codon, enabling the detection of gene-targeted polymorphisms [24]. Because these marker systems target different genomic regions and reveal complementary sources of genetic variation, their combined application increases genome coverage and improves the resolution of genetic diversity analyses compared with individual marker systems alone [28].

Although SSR markers remain highly informative and reproducible, their dependence on prior sequence information and species-specific primer development may limit their practicality for large-scale genetic diversity studies [21]. Nevertheless, SSRs have demonstrated strong utility for rice cultivar identification and genetic discrimination. Vemireddy et al. (2015) showed that a panel of only eight hypervariable SSR markers generated distinctive molecular fingerprints for 90 major Indian rice cultivars, demonstrating the potential of highly polymorphic markers for cultivar discrimination and DUS testing [29]. Aljumaili et al. (2018) likewise identified substantial genetic diversity and population structure among 50 Malaysian aromatic rice accessions using 32 SSR markers [30]. The complementary value of multiple marker systems has also been demonstrated in rice germplasm studies. Moonsap et al. (2019) reported improved discrimination of genetic diversity among Indo-China rice varieties through the combined use of ISSR, SRAP, and InDel markers [31], while Rini et al. (2023) demonstrated that integrating ISSR and SRAP markers enhanced the resolution of genetic diversity by capturing complementary patterns of genomic variation [28]. In addition, Saengprajak et al. (2024) confirmed the effectiveness of ISSR markers for characterizing Thai rice germplasm and detecting substantial polymorphism [27], whereas Susiyanti et al. (2021) demonstrated considerable genetic differentiation between aromatic and non-aromatic Indonesian rice varieties using SSR markers related to aromatic genes [32]. Collectively, these findings support the use of complementary molecular marker systems to provide broader and more robust resolution of genetic diversity than reliance on a single marker system.

Rice fragrance has long been attributed primarily to the accumulation of 2-acetyl-1-pyrroline (2AP), which results predominantly from loss-of-function mutations in the *BADH2* gene [33,34,35]. Beyond the established role of *BADH2*, genetic studies have indicated that rice aroma may involve additional genomic regions and candidate genes. By integrating QTL mapping with transcriptome profiling in aromatic and non-aromatic rice lines, Pachauri et al. (2014) identified differentially expressed candidate genes within aroma-associated QTL regions on chromosomes 3 and 4, together with *BADH2* on chromosome 8, suggesting a broader genetic basis underlying rice grain aroma [36]. More recently, Imran et al. (2023) comprehensively reviewed the molecular and biochemical mechanisms underlying 2AP biosynthesis, highlighting that aroma expression is regulated not only by *BADH2* but also by environmental conditions, agronomic practices, and interactions among multiple metabolic pathways [37]. However, increasing evidence indicates that rice aroma is a complex trait governed by the combined effects of numerous volatile organic compounds (VOCs), including aldehydes, alcohols, ketones, esters, furans, hydrocarbons, and nitrogen-containing compounds, whose composition and abundance are influenced by both genetic background and environmental conditions. Zheng et al. (2022) summarized more than 300 VOCs associated with cooked rice aroma and highlighted the application of gas chromatography-mass spectrometry (GC-MS), gas chromatography-olfactometry (GC-O), and other analytical approaches for comprehensive aroma characterization [38], whereas Kongpun et al. (2024) reviewed how genetic, environmental, agronomic, and postharvest factors collectively influence aroma formation and stability throughout the fragrant rice value chain [39]. Consistent with these findings, recent GC-MS-based metabolomic studies have revealed substantial variation in VOC profiles between aromatic and non-aromatic rice cultivars, reflecting differences in metabolic pathways and biochemical mechanisms underlying aroma formation [38,39,40,41].

Recent advances in plant genomics, metabolomics, and multi-omics integration have highlighted the value of combining genome-wide molecular fingerprinting with volatile metabolite profiling for comprehensive germplasm characterization. Such integrative approaches simultaneously reveal genetic diversity, biochemical variation, and genotype–phenotype relationships, providing deeper insights into germplasm resources than either molecular or metabolomic analyses alone [42,43]. In addition, multivariate statistical analyses enable the visualization of genetic structure, evaluation of molecular variation, and exploration of relationships between genetic diversity and metabolite composition, thereby supporting germplasm conservation, cultivar authentication, and parent selection in breeding programs [43,44]. Despite these advances, studies integrating complementary DNA marker systems with comprehensive volatile metabolite profiling in Thai aromatic rice remain limited. Most previous investigations have examined either molecular genetic diversity or aroma-related volatile composition independently, providing only partial insights into the genetic and biochemical mechanisms underlying rice aroma. Consequently, the relationship between multilocus molecular diversity and aroma-associated metabolite profiles among Thai aromatic rice cultivars remains insufficiently understood [41,45].

Therefore, the present study integrated complementary ISSR, SCoT, and SRAP molecular marker systems with static headspace GC–MS (HS-GC–MS) volatile profiling to characterize the genetic diversity and aroma-related biochemical variation in Thai aromatic and non-aromatic rice cultivars. Specifically, this study aimed to (i) evaluate molecular genetic diversity using complementary DNA marker systems, (ii) assess genetic relationships and clustering patterns among the cultivars, (iii) characterize representative volatile compounds in selected cultivars using HS-GC–MS, and (iv) explore differences in volatile profiles among the analyzed samples in the context of the genetic relationships revealed by molecular markers. This integrated approach provides complementary genetic and exploratory biochemical information for rice germplasm characterization, cultivar discrimination, and the selection of diverse materials for future rice breeding and germplasm management.

## 2. Results

### 2.1. Polymorphism and Discriminatory Efficiency of Molecular Markers

**Figure 1:**
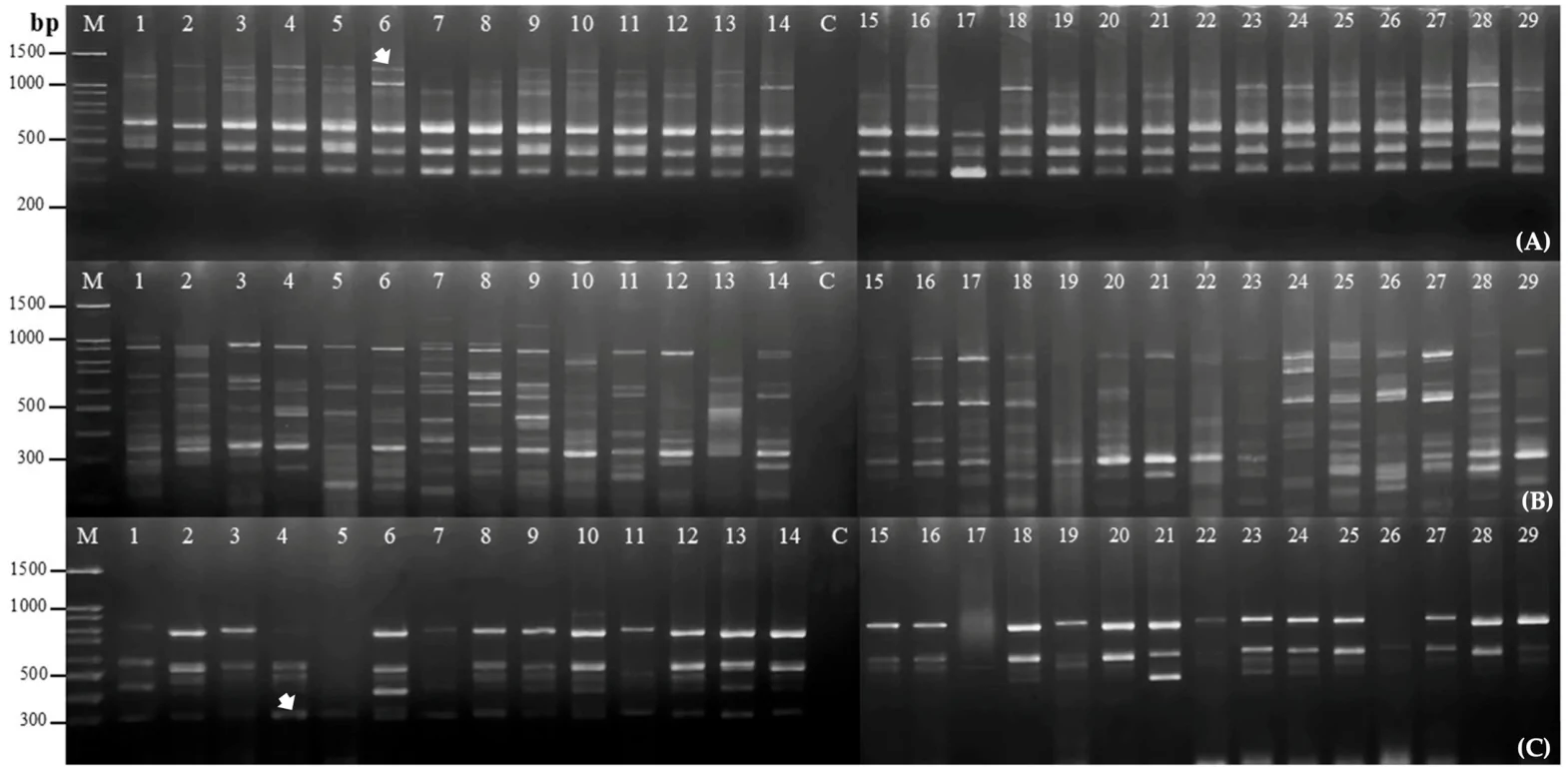
Representative DNA fingerprinting profiles of 29 Thai rice cultivars generated using (**A**) ISSR primer UBC818, (**B**) SCoT primer SCoT27, and (**C**) the SRAP primer combination Me4/Em5, separated on 1.5% (*w*/*v*) agarose gels. Lane M, 100-bp DNA ladder; lanes 1–29, Thai rice cultivars listed in Table 2; lane C, non-template control (NTC). Arrowheads indicate candidate bands associated with the aromatic cultivars examined, including a 1250-bp ISSR fragment amplified using primer UBC818 and a 300-bp SRAP fragment amplified using the Me4/Em5 primer combination. Both fragments were detected in aromatic cultivars but were absent from the non-aromatic cultivars examined in this study.

Genetic polymorphism among the 29 Thai rice cultivars, comprising 14 aromatic and 15 non-aromatic cultivars, was evaluated using three PCR-based marker systems: ISSR, SCoT, and SRAP. Representative amplification profiles generated by each marker system are shown in Figure 1, while amplification characteristics and marker efficiency parameters are summarized in Table 1. Marker performance was assessed based on the total number of amplified bands (NTB), number of polymorphic bands (NPB), percentage of polymorphic bands (PP), polymorphic information content (PIC), resolving power (RP), marker index (MI), expected heterozygosity (*H_e_*), and the presence of candidate bands associated with the aromatic cultivars examined.

ISSR markers. The ten ISSR primers generated 54 scorable bands, of which 20 were polymorphic, corresponding to a mean polymorphism of 35.07% (Table 1). Individual primers amplified 4–7 bands (mean = 5.40 bands per primer), with amplification products ranging from 200 to 1600 bp. Among the primers evaluated, UBC834 showed the highest PIC (0.208), RP (2.280), MI (1.250), and *H_e_* (0.262). In contrast, UBC859 produced only monomorphic bands and consequently showed no detectable polymorphism (PIC = RP = MI = *H_e_* = 0). The mean PIC, RP, MI, and *H_e_* values across the ten ISSR primers were 0.109, 1.159, 0.624, and 0.135, respectively. Notably, primer UBC818 amplified a 1250-bp fragment that was detected in the aromatic cultivars but absent from the non-aromatic cultivars examined (Table 1; Figure 1A). This fragment represents a candidate band associated with the aromatic cultivars examined and requires further validation in a broader collection of rice genotypes.

SCoT markers. The ten SCoT primers generated 54 amplified bands, including 28 polymorphic bands, corresponding to a mean polymorphism of 51.81% (Table 1). Individual primers amplified 4–7 bands (mean = 5.40 bands per primer), with amplification products ranging from 100 to 1600 bp. The percentage of polymorphism ranged from 33.33% for SCoT6 and SCoT29 to 80.00% for SCoT27. The mean PIC, RP, MI, and *H_e_* values were 0.142, 1.407, 0.771, and 0.176, respectively. Among the primers evaluated, SCoT27 showed the highest PIC (0.197), whereas SCoT17 showed the highest RP (2.280) and MI (1.350). SCoT17 also had the highest *H_e_* value (0.238), marginally exceeding that of SCoT27 (0.236). In contrast, SCoT21 showed the lowest PIC (0.061), RP (0.340), MI (0.300), and *H_e_* (0.065). Overall, SCoT markers detected a higher percentage of polymorphic bands and showed higher mean PIC and He values than ISSR markers in this collection.

SRAP markers. The ten SRAP primer combinations generated 62 amplified bands, of which 50 were polymorphic, corresponding to a mean polymorphism of 79.19% (Table 1). Individual primer combinations amplified 5–7 bands (mean = 6.20 bands per combination), with fragment sizes ranging from 100 to 1700 bp. The percentage of polymorphism ranged from 40.00% for Me1/Em2 to 100.00% for Me1/Em1 and Me5/Em1. Among the three marker systems, SRAP showed the highest mean PIC (0.214), RP (2.469), MI (1.357), and *H_e_* (0.267), indicating the greatest overall discriminatory efficiency based on these parameters. Among the primer combinations, Me1/Em1 showed the highest PIC (0.339), MI (2.040), and *H_e_* (0.434), while its RP (4.000) was the highest, together with Me5/Em1. In contrast, Me1/Em2 showed the lowest PIC (0.076), RP (0.480), MI (0.380), and *H_e_* (0.085). In addition, the Me4/Em5 primer combination amplified a 300-bp putative DNA fragment associated with aromatic cultivars (Table 1; Figure 1C). Overall, SRAP generated the highest percentage of polymorphic bands and showed the highest mean marker efficiency parameters among the three marker systems evaluated.

Comparison of marker systems. The three marker systems differed in their ability to detect genetic polymorphism among the 29 Thai rice cultivars (Table 1). SRAP generated the highest number of amplified bands (62) and polymorphic bands (50), as well as the highest mean polymorphism (79.19%) and mean values of PIC (0.214), RP (2.469), MI (1.357), and *H_e_* (0.267). SCoT showed intermediate performance, with 28 polymorphic bands and a mean polymorphism of 51.81%, whereas ISSR generated 20 polymorphic bands and a mean polymorphism of 35.07%. Nevertheless, ISSR and SRAP each revealed a candidate band associated with the aromatic cultivars examined: a 1250-bp ISSR fragment amplified using primer UBC818 and a 300-bp SRAP fragment amplified using the Me4/Em5 primer combination. Both fragments were detected in the aromatic cultivars and absent from the non-aromatic cultivars examined. Collectively, the three marker systems provided complementary information on genetic variation, with SRAP showing the highest overall marker informativeness among the three systems. The two fragments associated with aromatic rice cultivars represent candidate markers for further validation in broader rice germplasm collections.

**Table 2:** Characteristics of the Thai rice cultivars included in this study.

No.	Rice Cultivar	Accession No.	Origin	Aroma Type	Pericarp Color	Endosperm Type
1	Khao Gor Kor 6 (RD6)	21574	Nakhon Ratchasima	Aromatic	White	Glutinous
2	Khao Gor Kor 15 (RD15)	16006	Northeast Thailand	Aromatic	White	Non-glutinous
3	Khao Gor Kor 18 (RD18)	24854	Northeast Thailand	Aromatic	White	Glutinous
4	Khao Gor Kor 33 (RD33)	24550	Ubon Ratchathani	Aromatic	White	Non-glutinous
5	Khao Gor Kor 45 (RD45)	24608	Prachin Buri	Aromatic	White	Non-glutinous
6	Khao Gor Kor 77 (RD77)	24868	Pathum Thani	Aromatic	White	Non-glutinous
7	Khao Gor Kor 83 (RD83)	24871	Nong Khai	Aromatic	Purple-black	Non-glutinous
8	Khao Gor Kor 97 (RD97)	24924	Pathum Thani	Aromatic	White	Non-glutinous
9	Khao Dawk Mali 105 (KDML105)	12230	Chachoengsao	Aromatic	White	Non-glutinous
10	Khao Jao Hom Suphanburi	23062	Suphan Buri	Aromatic	White	Non-glutinous
11	Khao Niao RD6	21547	Northeast Thailand	Aromatic	White	Glutinous
12	Pathum Thani 1 (PTT1)	23898	Pathum Thani	Aromatic	White	Non-glutinous
13	Khao Mali Nin Surin	24872	Maha Sarakham	Aromatic	Purple-black	Glutinous
14	Khao Hom Bai Toey 62	24901	Nakhon Sawan, Uthai Thani	Aromatic	White	Non-glutinous
15	Khao Gor Kor 31 (RD31)	24533	Suphan Buri	Non-aromatic	White	Non-glutinous
16	Khao Gor Kor 41 (RD41)	24605	Chai Nat	Non-aromatic	White	Non-glutinous
17	Khao Gor Kor 43 (RD43)	24606	Suphan Buri	Non-aromatic	White	Non-glutinous
18	Khao Gor Kor 53 (RD53)	24858	Phrae	Non-aromatic	White	Non-glutinous
19	Khao Gor Kor 57 (RD57)	24860	Suphan Buri	Non-aromatic	White	Non-glutinous
20	Khao Gor Kor 61 (RD61)	24896	Chai Nat	Non-aromatic	Light brown	Non-glutinous
21	Khao Gor Kor 63 (RD63)	24862	Pathum Thani	Non-aromatic	White	Non-glutinous
22	Khao Gor Kor 71 (RD71)	24866	Phitsanulok	Non-aromatic	White	Non-glutinous
23	Khao Gor Kor 79 (RD79)	24869	Chai Nat	Non-aromatic	White	Non-glutinous
24	Khao Gor Kor 81 (RD81)	24870	Pathum Thani	Non-aromatic	White	Non-glutinous
25	Khao Gor Kor 85 (RD85)	24881	Phitsanulok	Non-aromatic	White	Non-glutinous
26	Khao Gor Kor 87 (RD87)	24897	Pathum Thani	Non-aromatic	White	Non-glutinous
27	Khao Gor Kor 91 (RD91)	24893	Pathum Thani	Non-aromatic	Light brown	Non-glutinous
28	Khao Gor Kor 95 (RD95)	24923	Chai Nat	Non-aromatic	White	Non-glutinous
29	Khao Gor Kor 101 (RD101)	24925	Pathum Thani	Non-aromatic	White	Non-glutinous

Note: Germplasm accession numbers (GS No.) correspond to records maintained by the Rice Department of Thailand. RD denotes cultivars officially released by the Rice Department. Aroma type, pericarp color, and endosperm type were classified according to the descriptors of the Standard Evaluation System for Rice (SES) developed by the International Rice Research Institute (IRRI) [46].

### 2.2. Genetic Relationships Among Thai Rice Cultivars Revealed by Combined Molecular Markers

**Figure 2:**
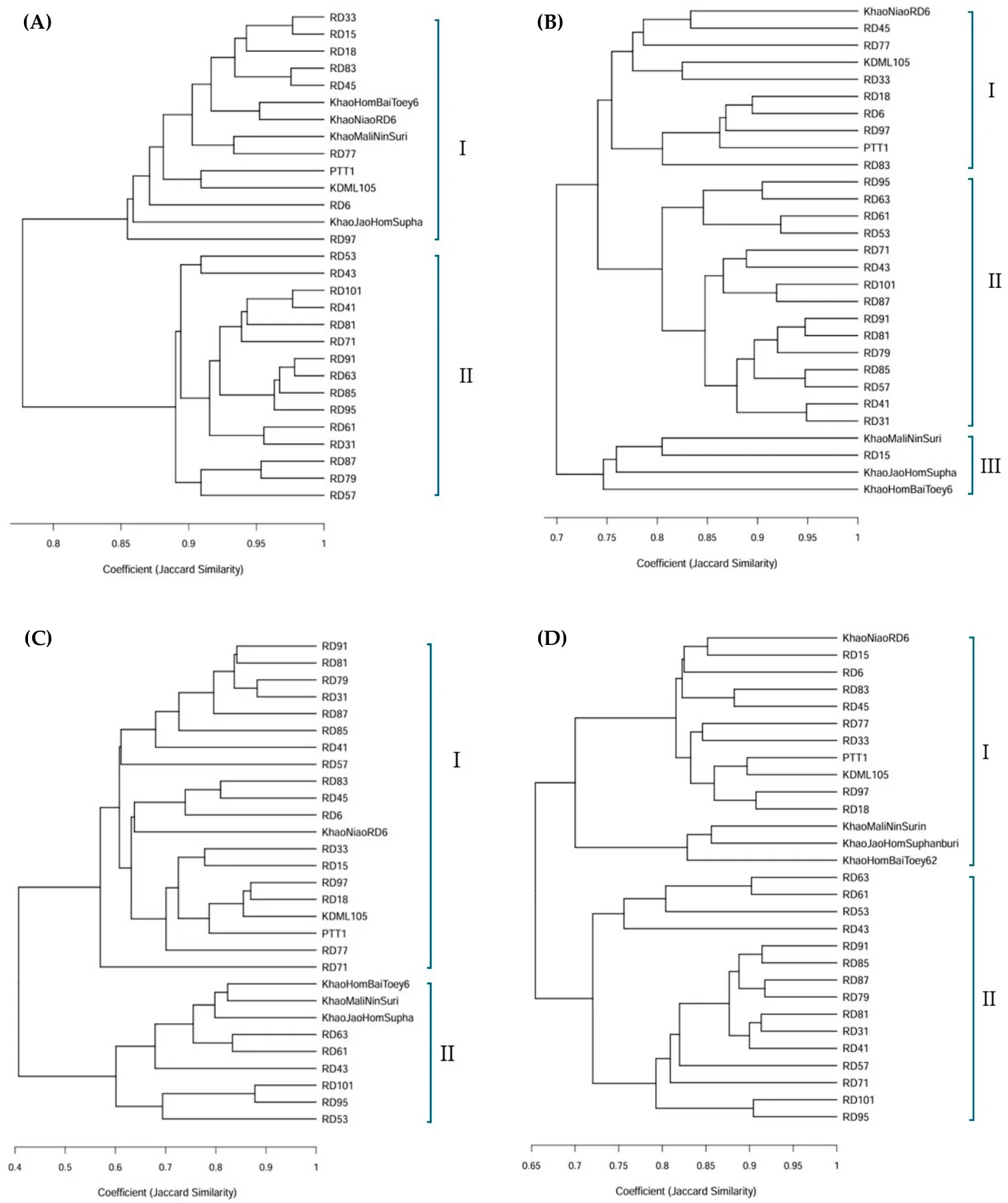
UPGMA dendrograms showing genetic relationships among 29 Thai rice cultivars based on (**A**) ISSR, (**B**) SCoT, (**C**) SRAP, and (**D**) combined ISSR, SCoT, and SRAP marker datasets. Genetic similarity was calculated using Jaccard’s similarity coefficient, and clustering was performed using the unweighted pair group method with arithmetic mean (UPGMA). Roman numerals (I, II, and III) indicate the major genetic clusters identified in each dendrogram.

Genetic relationships among the 29 Thai rice cultivars were evaluated using ISSR, SCoT, and SRAP markers individually and in combination. Binary marker profiles, scored according to the presence (1) or absence (0) of amplified fragments, were used to calculate pairwise genetic similarity coefficients based on Jaccard’s coefficient. The resulting similarity matrices were subjected to Unweighted Pair Group Method with Arithmetic Mean (UPGMA) clustering to characterize genetic relationships among the cultivars (Table 3; Figure 2A–D). The dendrograms derived from the individual marker systems exhibited differences in their capacity to resolve genetic relationships. ISSR markers produced relatively limited subdivision, whereas SCoT markers distinguished additional subgroups among some cultivars. SRAP markers yielded more differentiated clustering patterns, consistent with their comparatively higher PIC, RP, and MI values (Table 1; Figure 2). These differences indicate that the marker systems varied in their discriminatory performance and support the integration of complementary marker profiles for a more comprehensive assessment of genetic diversity. Supporting molecular diversity data, including the binary marker matrix (Table S1), Jaccard similarity coefficients (Table S2), and PCoA plot (Figure S1), are provided in the Supplementary Materials.

The combined ISSR, SCoT, and SRAP dataset resolved the 29 cultivars into two major genetic clusters (Figure 2D). Cluster I comprised the 14 cultivars classified as aromatic in Table 2: Khao Niao RD6, Khao Gor Kor 15 (RD15), Khao Gor Kor 6 (RD6), Khao Gor Kor 83 (RD83), Khao Gor Kor 45 (RD45), Khao Gor Kor 77 (RD77), Khao Gor Kor 33 (RD33), Pathum Thani 1 (PTT1), Khao Dawk Mali 105 (KDML105), Khao Gor Kor 97 (RD97), Khao Gor Kor 18 (RD18), Khao Mali Nin Surin, Khao Jao Hom Suphanburi, and Khao Hom Bai Toey 62. Cluster II contained the 15 cultivars classified as non-aromatic: Khao Gor Kor 31 (RD31), Khao Gor Kor 41 (RD41), Khao Gor Kor 43 (RD43), Khao Gor Kor 53 (RD53), Khao Gor Kor 57 (RD57), Khao Gor Kor 61 (RD61), Khao Gor Kor 63 (RD63), Khao Gor Kor 71 (RD71), Khao Gor Kor 79 (RD79), Khao Gor Kor 81 (RD81), Khao Gor Kor 85 (RD85), Khao Gor Kor 87 (RD87), Khao Gor Kor 91 (RD91), Khao Gor Kor 95 (RD95), and Khao Gor Kor 101 (RD101). The complete correspondence between the two major clusters and the recorded aroma classifications indicates that the combined molecular marker profiles effectively differentiated the aromatic and non-aromatic groups within the investigated germplasm collection. Nevertheless, this correspondence represents an association between multilocus genetic similarity and the existing cultivar classifications rather than evidence of a direct causal relationship between the detected markers and aroma.

The pairwise Jaccard similarity matrix further characterized the extent of molecular similarity among the cultivars (Table 3), with coefficients ranging from 0.54 to 0.92. The lowest similarity coefficient (0.54) was observed between Khao Gor Kor 45 (RD45; C5) and Khao Gor Kor 53 (RD53; C18), whereas the highest coefficient (0.92) occurred between Khao Gor Kor 79 (RD79; C23) and Khao Gor Kor 87 (RD87; C26). Khao Gor Kor 31 (RD31) and Khao Gor Kor 81 (RD81) also exhibited a high similarity coefficient, indicating closely related marker profiles. In contrast, the lower coefficients observed for other cultivar pairs reflected greater differentiation in their combined amplification patterns. This variation demonstrates that the germplasm collection contains both closely related cultivars and cultivars with more divergent molecular profiles, providing information on genetic variation at both the group and cultivar-pair levels beyond the broad separation into aromatic and non-aromatic groups. 

**Figure 3:**
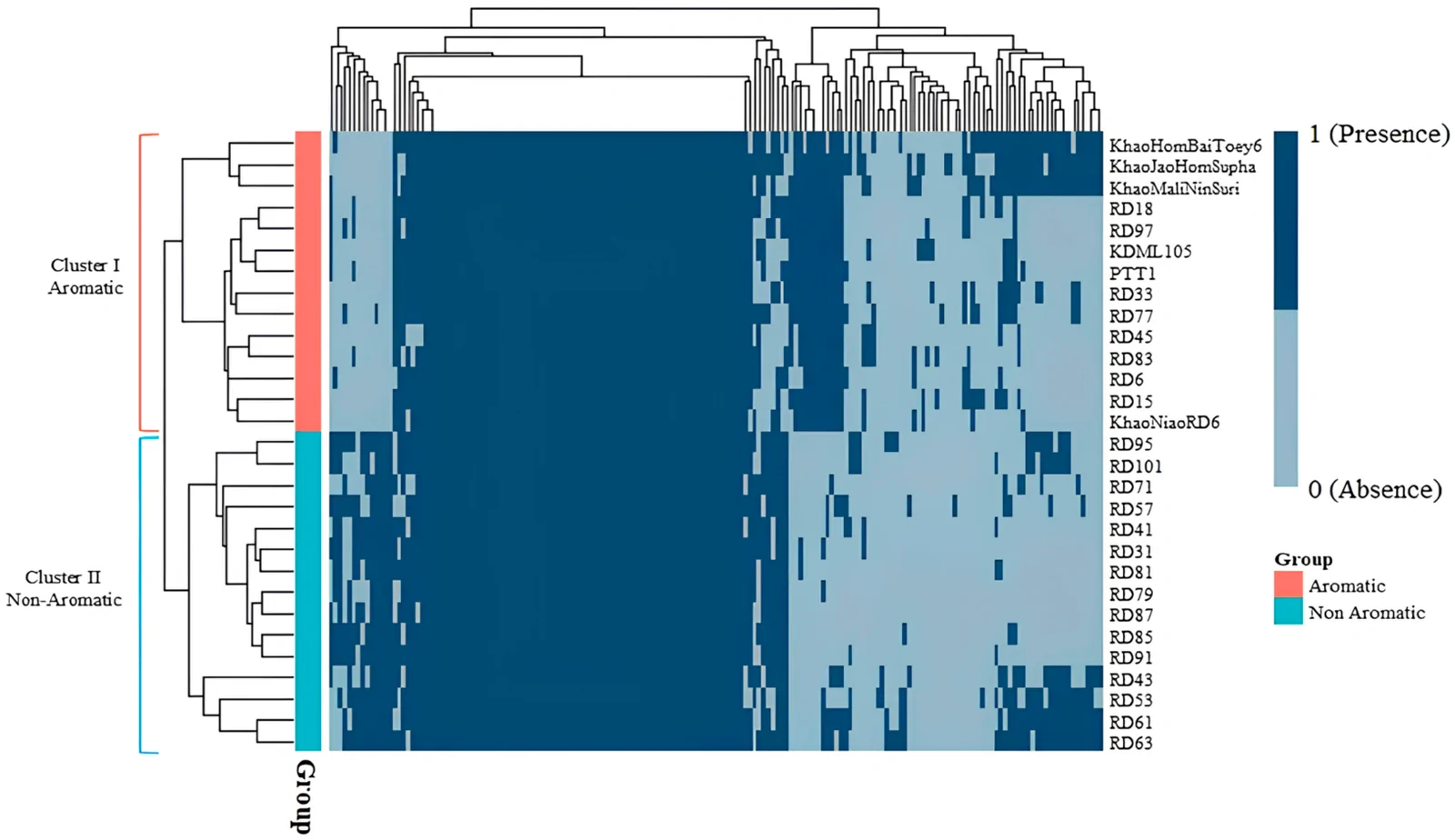
Hierarchical clustering heatmap of 29 Thai rice cultivars based on the combined ISSR, SCoT, and SRAP marker profiles. Rows represent rice cultivars, and columns represent binary-scored marker loci. Dark blue indicates the presence (1) of amplified fragments, whereas light blue indicates their absence (0). Hierarchical clustering was performed using the unweighted pair group method with arithmetic mean (UPGMA). The heatmap was generated in R version 4.3.2 using the pheatmap package.

The hierarchical clustering heatmap provided an independent visualization of the distribution of binary marker profiles among the 29 cultivars (Figure 3). The matrix displayed the presence and absence of amplified fragments across the scored loci, while hierarchical clustering organized cultivars according to similarities in their multilocus profiles. The resulting dendrogram separated the cultivars into Cluster I, comprising the aromatic group, and Cluster II, comprising the non-aromatic group, consistent with the combined-marker UPGMA dendrogram (Figure 2D) and the classifications reported in Table 2. Variation in fragment-presence and fragment-absence patterns was evident across the collection, reflecting molecular differences both within and between the two groups. The agreement between the heatmap and UPGMA analysis strengthens confidence in the observed clustering pattern because the same genetic structure was evident in complementary representations of the combined marker data. However, the separation of the groups should be interpreted in terms of overall marker-profile similarity rather than as evidence that amplified fragments are responsible for aroma characteristics.

**Figure 4:**
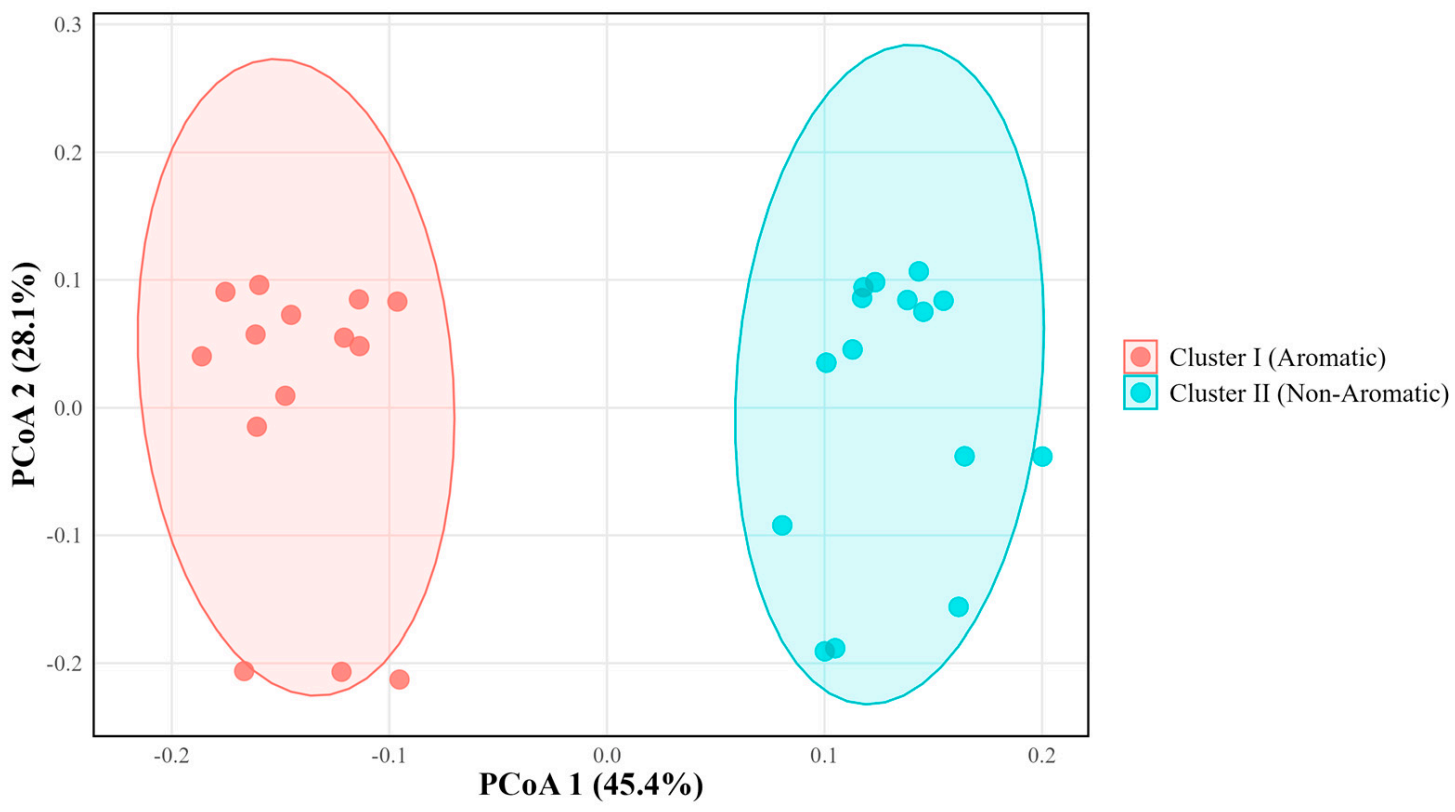
Principal coordinate analysis (PCoA) of 29 Thai rice cultivars based on the combined ISSR, SCoT, and SRAP marker dataset. Genetic distances were derived from Jaccard’s similarity coefficients. The scatter plot shows the relationships among cultivars along the first two principal coordinates (PCoA1 and PCoA2). The analysis was performed using R version 4.3.2.

Principal coordinate analysis (PCoA) further illustrated the genetic relationships among the 29 cultivars using the combined ISSR, SCoT, and SRAP dataset (Figure 4). The ordination plot showed a distinct distribution of the aromatic and non-aromatic cultivars, broadly consistent with the groupings obtained through UPGMA clustering and hierarchical heatmap analysis. According to the percentages displayed in Figure 4, the first principal coordinate (PCoA1) accounted for 37.2% of the variation, while the second principal coordinate (PCoA2) accounted for 27.1%, together representing 64.3% of the variation in the first two coordinates. The aromatic cultivars were generally positioned toward the negative side of PCoA1, whereas the non-aromatic cultivars were predominantly distributed toward the positive side. The separation of the group confidence ellipses further illustrated differences between the molecular profiles of the two aroma groups. The consistency between the ordination and clustering results indicates that the observed differentiation was not restricted to a single hierarchical representation but was also evident in the multidimensional relationships among the cultivars. Collectively, these analyses demonstrate that the combined molecular marker dataset captured genetic structure corresponding to the recorded aroma classifications within this collection.

**Figure 5:**
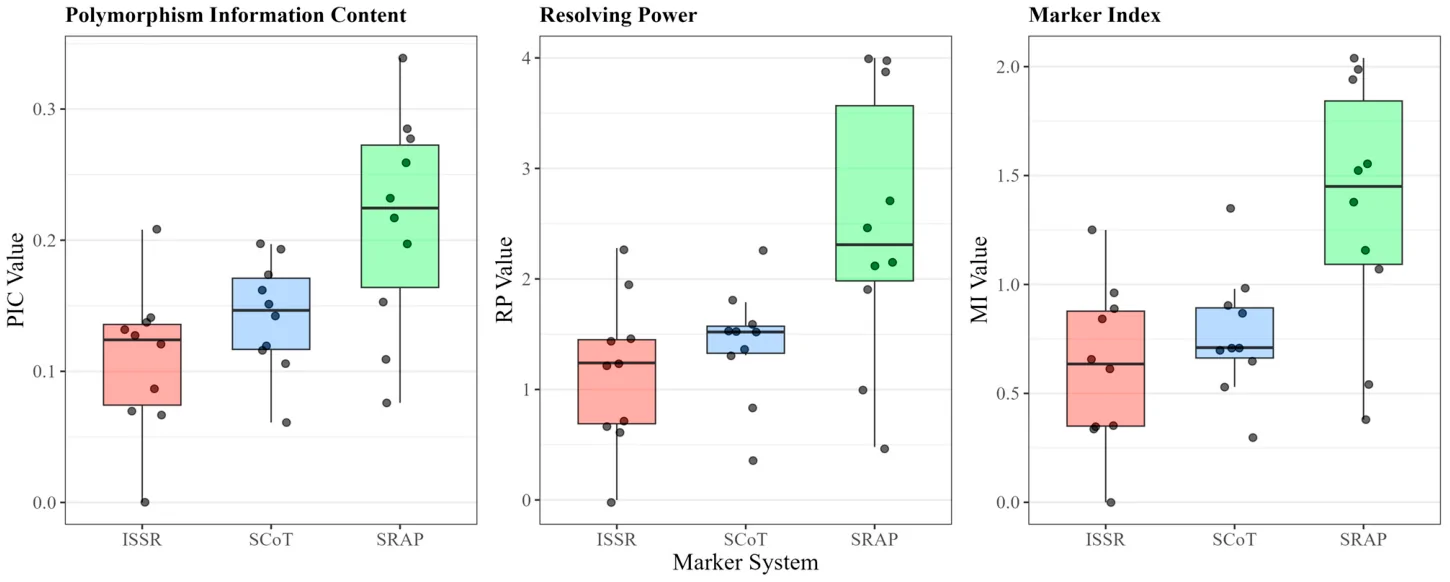
Comparison of marker efficiency among ISSR, SCoT, and SRAP marker systems used to evaluate genetic diversity in 29 Thai rice cultivars. Boxplots show the distributions of PIC, RP, and MI values for each marker system. The horizontal black line within each box represents the median, while the lower and upper edges of the box represent the first and third quartiles, respectively. Individual black dots represent values obtained from individual primers or primer combinations. Different colors represent ISSR (pink), SCoT (blue), and SRAP (green). The analyses were performed using R software (version 4.3.2) with the ggplot2 package.

The comparative distributions of PIC, RP, and MI values further clarified the relative performance of the three marker systems (Figure 5). SRAP exhibited the highest median values for all three parameters, followed by SCoT and ISSR, in agreement with the numerical estimates reported in Table 1. The higher PIC values indicated greater marker informativeness, while the higher RP and MI values reflected greater discriminatory power and overall marker efficiency under the conditions of this study. The broader distributions observed for SRAP also indicated greater variation in the performance of individual primers or primer combinations. SCoT showed intermediate values, whereas ISSR generally exhibited lower median values across the three parameters. These findings demonstrate that the effectiveness of individual marker systems differed in their ability to reveal polymorphism and distinguish among the investigated cultivars. Although SRAP showed the strongest performance across the evaluated efficiency parameters, combining ISSR, SCoT, and SRAP provided an integrated molecular dataset for examining genetic relationships. The concordant patterns observed in the UPGMA dendrogram, hierarchical heatmap, and PCoA collectively support the utility of this combined approach for characterizing genetic diversity and differentiating Thai rice germplasm according to the aroma classifications recorded in Table 2.

### 2.3. Identification and Comparative Analysis of Volatile Compounds by HS-GC–MS

Based on the genetic relationships inferred from the combined ISSR, SCoT, and SRAP marker dataset (Figure 2D), four representative Thai rice cultivars, KDML105, PTT1, RD43, and RD79, were selected for comparative volatile profiling. The selection was guided by the molecular clustering pattern to capture genetic variation within the rice germplasm. KDML105 and PTT1 represented aromatic cultivars from distinct branches within the aromatic group, whereas RD43 and RD79 represented non-aromatic cultivars from separate subclusters. Thus, the selected cultivars encompassed both inter-group variation between aromatic and non-aromatic rice and intra-group genetic variation, providing a basis for an exploratory comparison of volatile profiles in cooked rice.

**Figure 6:**
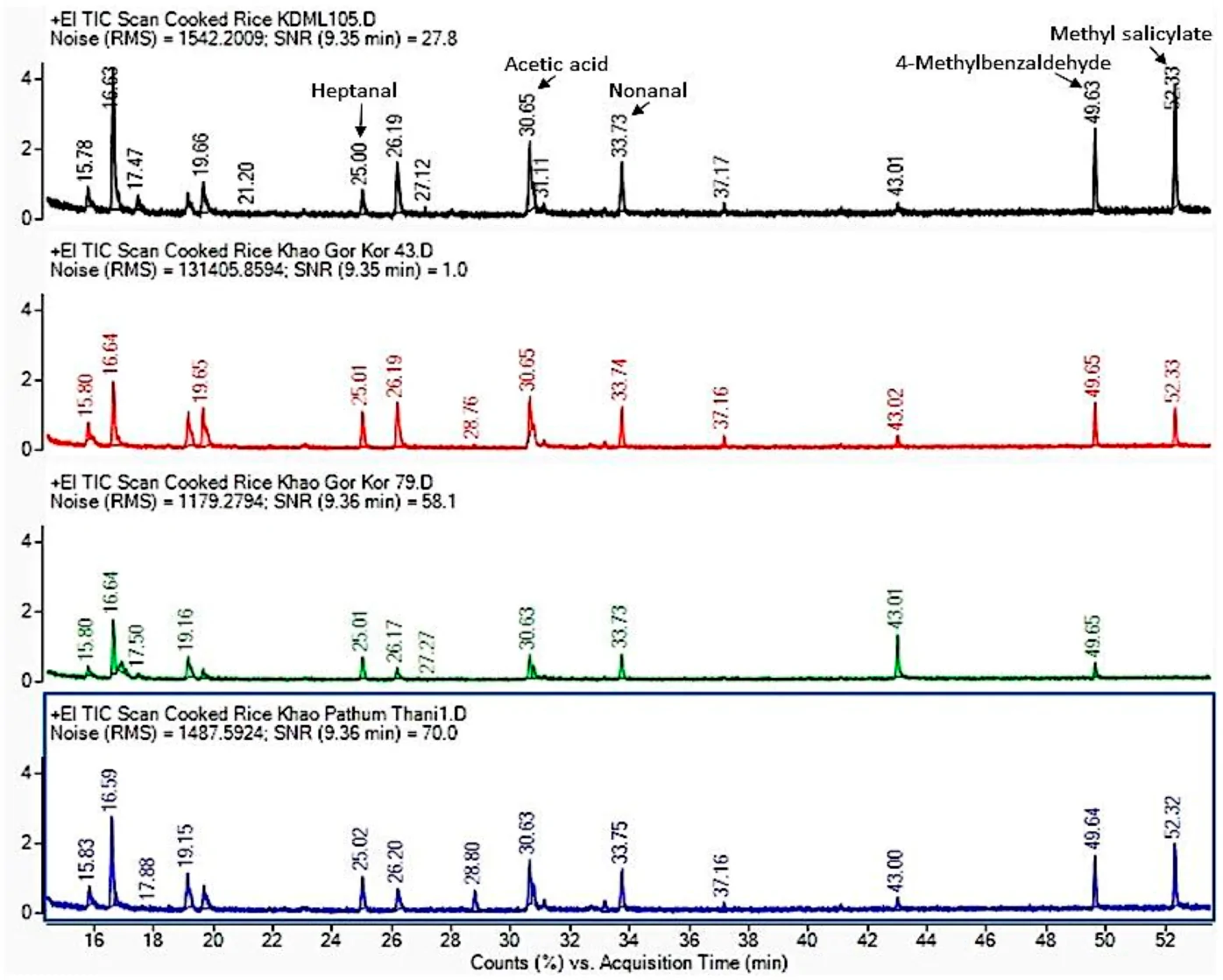
Total ion chromatograms (TICs) of volatile compounds detected in cooked rice cultivars (KDML105, RD43, RD79, and PTT1) using static headspace gas chromatography–mass spectrometry (HS-GC–MS). The chromatograms are presented in black (KDML105), red (RD43), green (RD79), and blue (PTT1). The six tentatively identified compounds, namely hexanal, heptanal, acetic acid, nonanal, 4-methylbenzaldehyde, and methyl salicylate, are annotated in the chromatogram of KDML105 as representative examples.

Representative total ion chromatograms (TICs) obtained by HS-GC–MS are shown in Figure 6. The four cultivars exhibited broadly comparable chromatographic patterns, with differences in peak intensities and relative abundances indicating variation in the observed volatile profiles. Six volatile compounds were tentatively identified by comparing their mass spectra with reference spectra in the NIST23 and Wiley23 mass spectral libraries using Agilent MassHunter Qualitative Analysis software (Table 4). Library match values ranged from 71.43% to 90.85%, and no predefined minimum library-matching threshold was applied. Retention indices were compared with published reference values to provide additional support for compound annotation. The six compounds comprised four aldehydes (hexanal, heptanal, nonanal, and 4-methylbenzaldehyde), one carboxylic acid (acetic acid), and one ester (methyl salicylate). Although authentic reference standards were not used for confirmation, spectral library matching and comparison with published retention indices provided a basis for tentative compound identification.

Hexanal showed the highest relative abundance among the six reported compounds in all four cultivars, accounting for 19.51–51.30% of the total chromatographic peak area. RD79 exhibited the highest observed relative abundance of hexanal (51.30%), followed by PTT1 (49.64%) and RD43 (40.79%), whereas KDML105 showed a lower proportion (19.51%). Heptanal and nonanal were detected in all four cultivars at relatively low abundances, ranging from 0.89–2.02% and 1.33–1.87%, respectively. Among the other reported compounds, acetic acid showed an observed relative abundance of 3.22% in KDML105, compared with 1.14–1.97% in the other three cultivars. Similarly, 4-methylbenzaldehyde had observed relative abundances of 2.47% in KDML105 and 0.98% in RD79. Methyl salicylate showed an observed relative abundance of 3.55% in KDML105, whereas no corresponding peak was detected in RD79 under the analytical conditions used.

Overall, HS-GC–MS profiling provided preliminary insights into the relative abundances of six tentatively identified volatile compounds among the four selected Thai rice cultivars. Aldehydes represented the predominant chemical class among the reported compounds, with hexanal showing the highest relative abundance in all four samples. Acetic acid, 4-methylbenzaldehyde, and methyl salicylate also exhibited distinct patterns of relative abundance across the analyzed samples. These findings highlight the potential of HS-GC–MS profiling as a complementary approach to characterizing volatile composition in rice and provide a foundation for exploratory multivariate analysis alongside molecular marker data. Although the results are descriptive because independent biological replication was not performed, the observed patterns offer a useful starting point for further investigation.

### 2.4. Multivariate Analysis of Volatile Compounds

**Figure 7:**
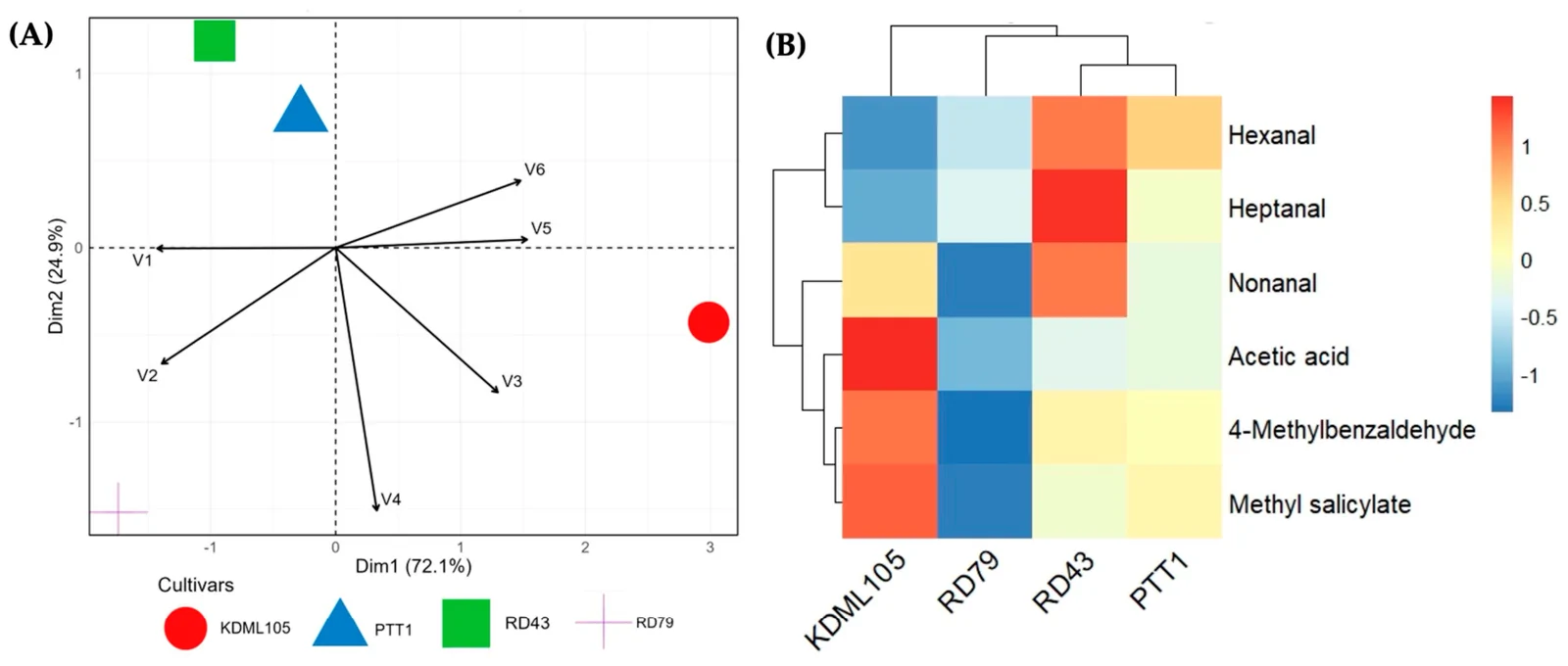
Multivariate analysis of volatile compounds detected in cooked rice samples (KDML105, RD43, RD79, and PTT1) using HS-GC–MS. (**A**) PCA biplot based on the relative peak areas (%) of six tentatively identified volatile compounds, using variable-wise (compound-wise) standardization. (**B**) Hierarchical clustering heatmap based on compound-wise Z-score-standardized relative peak areas (%) using Euclidean distance and Ward.D2 linkage. Columns represent rice cultivars, and rows represent volatile compounds. Colors show compound-wise Z-scores of relative peak areas (%), with red indicating values above and blue indicating values below the mean for each compound across the four analyzed samples. Figure 7B was regenerated from the percentage data in Table 3. The dendrograms illustrate exploratory clustering patterns among the cultivars and volatile compounds based on their standardized relative profiles.

Principal component analysis (PCA) and hierarchical clustering analysis (HCA) were performed using the relative peak areas of six representative volatile compounds measured in four selected rice cultivars (KDML105, PTT1, RD43, and RD79) by HS-GC–MS to explore patterns of variation in their volatile profiles (Figure 7). The first two principal components accounted for 97.0% of the total variance, with Dim1 and Dim2 explaining 72.1% and 24.9%, respectively (Figure 7A). KDML105 was positioned on the positive side of Dim1, whereas PTT1, RD43, and RD79 were located on the negative side. This pattern indicated a clear separation of KDML105 within the dataset, particularly along Dim1. Acetic acid (V3), 4-methylbenzaldehyde (V5), and methyl salicylate (V6) were associated with the positive direction of Dim1, whereas hexanal (V1) and heptanal (V2) were associated with the negative direction. Nonanal (V4) was primarily associated with the negative direction of Dim2. Dim2 provided additional differentiation among the cultivars, particularly between RD79 and RD43/PTT1. These patterns suggest that differences in the relative peak areas of individual compounds contributed to the observed differentiation among the four cultivars.

HCA revealed a clustering pattern broadly consistent with the PCA results (Figure 7B). RD43 and PTT1 clustered closely and were subsequently grouped with RD79, whereas KDML105 formed a separate branch. The heatmap further illustrated differences in relative peak areas among the analyzed samples. KDML105 showed comparatively higher relative peak areas for acetic acid, tentatively annotated 4-methylbenzaldehyde, and methyl salicylate, whereas RD79 showed the highest relative peak areas for hexanal, heptanal, and nonanal. PTT1 also exhibited a relatively high peak area for hexanal, while RD79 showed the lowest relative peak areas for 4-methylbenzaldehyde and methyl salicylate. Together, PCA and HCA provided complementary exploratory views of variation in the observed volatile profiles, with KDML105 showing the most distinct pattern among the four analyzed samples. Nevertheless, these findings should be interpreted as exploratory because the analysis was based on only four cultivars and six tentatively annotated compounds, without independent biological replication. Thus, the observed patterns characterize the dataset examined and require validation using a larger cultivar panel and independently replicated samples before broader conclusions can be drawn about volatile variation in Thai rice.

## 3. Discussion

Understanding genetic diversity together with aroma-associated biochemical variation is important for rice germplasm characterization, conservation, and breeding [12,20,38]. Molecular markers provide complementary information on genetic relationships and diversity among rice cultivars, whereas volatile metabolite profiling provides information on biochemical variation associated with grain quality, aroma, and consumer-related characteristics [5,6,38,47,48]. Previous studies have demonstrated the value of genomics-assisted approaches and metabolomics for characterizing crop genetic resources and investigating variation in complex traits [20,37,42]. In the present study, ISSR, SCoT, and SRAP markers were combined with HS-GC–MS-based volatile profiling to characterize Thai rice cultivars. Rather than relying on a single analytical approach, this integrated framework enabled molecular genetic diversity to be compared with differences in volatile composition among representative cultivars.

### 3.1. Complementary Molecular Markers Reveal Different Components of Genetic Diversity

The comparative performance of ISSR, SCoT, and SRAP markers demonstrated that the capacity to resolve genetic variation differed among marker systems, highlighting the value of a complementary multilocus approach to rice germplasm characterization. SRAP exhibited the highest median values of PIC, RP, and MI, followed by SCoT and ISSR (Table 1; Figure 5). These differences indicate that SRAP provided greater overall marker informativeness and discriminatory power under the conditions of this study, consistent with previous reports of its utility in assessing genetic diversity in rice germplasm [24,28]. Nevertheless, the relative performance of a marker system depends on the polymorphism detected within the particular germplasm collection and the primers employed. Integrating ISSR, SCoT, and SRAP therefore offers a broader assessment of molecular variation by combining the discriminatory contributions of different marker systems, rather than assuming that a single system adequately represents the genetic diversity of the entire collection [24,25,28].

The combined marker dataset provided a coherent representation of genetic relationships among the 29 Thai rice cultivars. UPGMA clustering resolved two major groups, and the corresponding patterns observed in the hierarchical clustering heatmap and PCoA supported the differentiation revealed by the combined binary marker profiles (Figure 2D, Figure 3 and Figure 4). The concordance among these analytical approaches strengthens the interpretation of the major grouping pattern because the differentiation was evident in both hierarchical relationships and multivariate ordination. This finding is consistent with previous studies demonstrating the value of integrating molecular marker systems for rice germplasm characterization [28,31]. The combined analysis integrated information from the three marker systems, providing a broader basis for characterizing genetic relationships among the 29 Thai rice cultivars and distinguishing major groups within the germplasm collection.

A notable finding was the complete correspondence between the two major molecular clusters and the aroma classifications recorded in Table 2. Cluster I comprised all 14 aromatic cultivars, whereas Cluster II contained all 15 non-aromatic cultivars. This pattern indicates that, within the present collection, the combined marker profiles discriminated consistently between cultivars assigned to the two aroma categories. However, this correspondence requires careful biological interpretation. Rice fragrance is strongly associated with functional variation at the *BADH2* locus, whereas ISSR, SCoT, and SRAP markers detect polymorphisms across multiple genomic regions without necessarily targeting genes involved in aroma biosynthesis [34,35,37]. Consequently, the correspondence between molecular clustering and aroma classification does not establish that the amplified loci are functionally involved in fragrance determination or are linked to *BADH2*. The observed pattern may reflect genetic structure within this particular collection, including broader differences among cultivars that coincide with their recorded aroma classifications. Distinguishing these possibilities would require targeted genotyping of aroma-associated loci and validation across additional germplasm with independently established aroma phenotypes.

The ISSR fragment amplified by primer UBC818 and the SRAP fragment generated by the Me4/Em5 primer combination represent potential candidates for further investigation in aromatic rice germplasm. At present, however, their application as diagnostic markers remain preliminary because the fragments have not been sequenced, and their association with aroma has not been independently validated. Establishing their molecular identity, determining whether they are reproducibly associated with aroma classification, and assessing their discriminatory performance across genetically diverse rice collections would be necessary before considering their use in marker-assisted selection. These steps are particularly important for dominant markers, for which fragment presence alone does not establish the identity of the underlying sequence or demonstrate a functional relationship with the trait of interest.

From a breeding perspective, the combined ISSR, SCoT, and SRAP approach provides a practical foundation for preliminary germplasm differentiation and the identification of genetically distinct materials for subsequent evaluation. Nevertheless, the dominant nature of these marker systems limits the resolution of allelic variation because heterozygous and homozygous individuals carrying the same detectable band cannot generally be distinguished. Higher-resolution approaches, including SNP, genotyping, genotyping-by-sequencing (GBS), and whole-genome resequencing, could overcome some of these limitations by providing more detailed information on genome-wide variation and genetic relationships [20,23]. In conjunction with targeted analysis of *BADH2* and other relevant genomic regions, these approaches could help determine whether the observed correspondence between molecular clusters and aroma classifications reflects underlying population structure, trait-associated variation, or both. Thus, the present findings establish the value of complementary dominant marker systems for characterizing Thai rice germplasm while identifying clear priorities for subsequent sequence-based validation and the development of reliable molecular tools for rice quality improvement.

### 3.2. Genetic Relationships Among Thai Rice Cultivars Revealed by Molecular Marker Systems

The combined ISSR, SCoT, and SRAP dataset resolved the 29 Thai rice cultivars into two major genetic clusters that corresponded to their recorded aroma classifications, with all 14 aromatic cultivars assigned to Cluster I and all 15 non-aromatic cultivars assigned to Cluster II. The concordance among UPGMA clustering, hierarchical clustering, and PCoA supports the consistency of this genetic differentiation across complementary analytical approaches. These findings demonstrate the utility of combining marker systems to characterize genetic relationships within the investigated germplasm collection. Because ISSR, SCoT, and SRAP markers provide complementary multilocus information, their integration offers a broader assessment of molecular variation than reliance on an individual marker system [21,28,31]. Similar applications of combined marker systems have demonstrated their value for resolving genetic diversity and relationships among rice germplasm [28,31].

The correspondence between molecular clustering and aroma classification raises the possibility that the observed genetic structure reflects, at least in part, the breeding histories and genetic backgrounds of the cultivars. Thai Hom Mali rice, particularly KDML105, has played an important role in fragrant rice production and breeding in Thailand [8]. Shared ancestry or the use of related aromatic germplasm could therefore contribute to similarities among some fragrant cultivars. Nevertheless, the present study did not investigate pedigree relationships or reconstruct breeding histories, and the observed clustering cannot establish that aromatic cultivars share a particular ancestral background. Furthermore, the separation of aromatic and non-aromatic cultivars should be interpreted as a pattern specific to the 29 cultivars examined rather than evidence that aroma classification universally determines genome-wide genetic relationships in Thai rice.

The correspondence observed in the combined-marker analysis also requires distinction between overall molecular relatedness and the genetic determinants of fragrance. Rice aroma is strongly associated with functional variation at the *BADH2* locus, whereas the ISSR, SCoT, and SRAP markers used in this study survey polymorphisms across multiple genomic regions [34,35,37]. Consequently, the observed association between multilocus marker profiles and aroma classification does not demonstrate that the amplified fragments are linked to *BADH2* or directly contribute to fragrance. Variation at aroma-associated loci may occur within broader genetic backgrounds, while cultivars with similar aroma classifications may differ elsewhere in their genomes. These distinctions between genome-wide diversity and trait-specific genetic variation have also been recognized in previous rice germplasm studies [10,30,37]. In the present collection, however, the complete correspondence between the two major clusters and the recorded aroma categories means that there was no observed exception requiring interpretation as a non-aromatic cultivar nested within the aromatic cluster.

The Jaccard similarity coefficients reported in Table 3 further characterize the molecular relationships among the cultivars, indicating variation in the degree of similarity between cultivar pairs. These differences provide a basis for identifying materials with distinct marker profiles for germplasm management and preliminary parental selection. Genetically differentiated cultivars may offer complementary genetic backgrounds for breeding, although their actual breeding value depends on the inheritance, diversity, and expression of the target traits rather than molecular distance alone [20]. The combined marker dataset can therefore support the preliminary characterization of Thai rice germplasm and the identification of materials for subsequent evaluation. However, the dominant nature of ISSR, SCoT, and SRAP markers limits allele-level interpretation because band presence generally does not distinguish heterozygous from homozygous genotypes. Higher-resolution approaches, including SNP genotyping, genotyping-by-sequencing, and whole-genome sequencing, could provide more detailed resolution of genetic relationships and population structure and facilitate the investigation of genomic regions associated with agronomic and grain-quality traits [20,23]. Integrating these approaches with targeted analysis of aroma-associated loci would help clarify the genetic basis of the observed correspondence between molecular clustering and aroma classification and strengthen the application of molecular information in Thai rice conservation and breeding.

### 3.3. Biological Significance of Volatile Profiles in Thai Rice Cultivars

Volatile compounds are important contributors to cooked rice aroma and consumer perception. Although more than 300 volatile compounds have been reported in rice, only a relatively small subset is considered to contribute substantially to aroma because their sensory impact depends on both odor threshold and abundance [38,47]. In the present study, HS-GC–MS analysis yielded six tentatively annotated volatile compounds belonging to the aldehyde, carboxylic acid, and ester classes. Their relative peak areas revealed preliminary variation in volatile profiles among the four cultivars.

Hexanal was the predominant volatile compound across the four cultivars, with the highest observed relative abundances in RD79 and PTT1. Hexanal is a major product of lipid oxidation, particularly through the lipoxygenase-mediated oxidation of linoleic acid, and is widely recognized as an important lipid-derived aldehyde associated with green and grassy notes in cooked rice [38,47]. The observed variation in hexanal relative abundance may reflect differences in lipid oxidation and related biochemical processes, although environmental conditions, grain storage, and post-harvest processing may also influence volatile composition. Other lipid-derived aldehydes, including heptanal and nonanal, were detected in all four cultivars, with relatively narrow ranges of observed relative abundance.

In contrast to the predominance of hexanal, KDML105 exhibited a distinct volatile profile, characterized by comparatively higher relative peak areas of acetic acid, 4-methylbenzaldehyde, and methyl salicylate. In particular, 4-methylbenzaldehyde and methyl salicylate have been reported among minor volatile constituents associated with the aroma of fragrant rice [38,49]. However, their relative abundances alone cannot establish their contribution to the characteristic aroma of KDML105, because odor activity values and sensory evaluation were not determined.

The characteristic aroma compound 2AP, an important contributor to fragrant rice aroma, was not detected under the analytical conditions used. However, its non-detection does not confirm its absence, as detection depends on factors such as concentration, headspace partitioning, and analytical sensitivity [47,51,52]. The six tentatively identified compounds provide complementary insights into volatile diversity among Thai rice cultivars. Further targeted quantification of 2AP and sensory evaluation would enhance understanding of the biochemical basis of rice aroma.

Multivariate analyses revealed variation in volatile profiles among the four analyzed rice samples. PCA separated the KDML105 sample from the other samples, while hierarchical clustering grouped the RD43 and PTT1 samples closely, followed by RD79. The distinct profile of KDML105 was characterized by comparatively higher relative peak areas of acetic acid, 4-methylbenzaldehyde, and methyl salicylate. These findings highlight the value of integrating volatile profiling and multivariate analysis with molecular marker-based characterization to provide complementary insights into biochemical diversity among rice cultivars. Consistent with previous metabolomic studies, the observed patterns suggest that rice volatile profiles reflect the combined contributions of multiple compounds and biochemical pathways [38,53]. Although preliminary because of the limited number of cultivars and absence of independent biological replication, these findings provide a useful initial characterization of volatile profile diversity in Thai rice.

Overall, integrating volatile profiling with molecular marker analysis provides a complementary approach to characterizing Thai rice germplasm. The distinct volatile profile of KDML105 and the predominance of hexanal across all four cultivars highlight the potential of volatile composition as an additional biochemical characteristic for cultivar differentiation. Although these findings are preliminary, they provide a basis for further investigating relationships between genetic diversity and aroma-related traits. Future studies incorporating larger germplasm collections, independent biological replication, quantitative analysis of key aroma compounds such as 2AP, and sensory evaluation could deepen understanding of rice aroma variation and support quality-oriented rice improvement.

### 3.4. Relationship Between Genetic Diversity and Volatile Metabolite Composition

Genome-wide molecular diversity and volatile metabolite composition represent complementary dimensions of variation in rice germplasm, providing insights into genetic relationships and aroma-associated biochemical characteristics, respectively. In the present study, ISSR, SCoT, and SRAP markers were used to characterize genetic relationships among 29 Thai rice cultivars, whereas HS-GC–MS profiling was restricted to four representative cultivars selected according to the combined molecular marker results. The molecular and volatile analyses revealed distinct patterns of variation among the selected cultivars, but their grouping patterns were not completely concordant. This observation suggests that differences in genome-wide molecular backgrounds do not necessarily translate directly into corresponding differences in volatile composition. Similar patterns of genetic and metabolite variation have been reported in studies integrating molecular or genomic data with metabolomic profiling, in which metabolite phenotypes reflect the combined influences of multiple genetic loci and biochemical pathways rather than individual molecular markers [37,42].

Several biological factors may contribute to differences between molecular relationships and volatile composition. ISSR, SCoT, and SRAP markers characterize multilocus genetic variation, but the amplified fragments examined in this study were not demonstrated to be directly associated with volatile biosynthesis. In contrast, volatile compounds represent downstream metabolic products arising from interconnected biochemical pathways, including lipid oxidation, amino acid metabolism, and phenylpropanoid metabolism, which may also be influenced by physiological conditions, environmental variation, and post-harvest processing [23,54]. Consequently, differences in volatile profiles cannot be attributed directly to genome-wide molecular divergence on the basis of the present data. Rather, the results illustrate the complementary nature of molecular marker analysis and volatile profiling, which characterize different aspects of rice diversity.

Six volatile compounds were tentatively identified across the four analyzed samples, with no corresponding methyl salicylate peak detected in RD79 under the analytical conditions used. Relative peak areas differed among the samples. KDML105 exhibited relatively higher proportions of acetic acid, 4-methylbenzaldehyde, and methyl salicylate, whereas RD79 showed the highest relative abundance of hexanal. Hexanal was the predominant compound across all four cultivars, accounting for 19.51–51.30% of the total chromatographic peak area, with the highest proportion detected in RD79 (51.30%) and the lowest in KDML105 (19.51%). These differences indicate that variation in relative peak areas contributed to the differentiation of volatile profiles among the selected cultivars. Accordingly, PCA and hierarchical clustering reflected differences in the relative composition of the detected compounds with non-detection of methyl salicylate in RD79 also contributing to the observed pattern. However, these profiles provide a preliminary characterization of volatile variation and should not be interpreted as a comprehensive representation of aroma, which depends on compound-specific odor thresholds, interactions among volatiles, and sensory perception.

Although the analysis was limited to four cultivars and six tentatively identified compounds, it provides an initial insight into variation in the volatile composition of Thai rice. Relative GC–MS peak areas provide semi-quantitative estimates and do not independently establish absolute compound concentrations or sensory contributions. The observed patterns therefore provide a basis for further investigating potential relationships between genetic diversity and volatile composition without establishing a direct genetic–metabolite association. Future studies incorporating larger and more genetically diverse rice populations, comprehensive volatile profiling, independent biological replication, targeted quantification of key aroma compounds, and sensory evaluation could clarify the relationships among genetic variation, volatile composition, and perceived aroma, supporting a more integrated understanding of rice quality and aroma-related traits.

### 3.5. Implications for Rice Breeding and Germplasm Conservation

The integrated analytical framework used in this study provides complementary information for rice germplasm characterization and breeding. The combined ISSR, SCoT, and SRAP marker dataset revealed genetic variation among the Thai rice cultivars and identified genetically differentiated accessions within the germplasm collection. Such information can complement conventional phenotypic evaluation by providing an additional basis for assessing genetic relationships and identifying diverse germplasm for breeding programs. Genetic diversity information derived from molecular markers may therefore contribute to parental selection when considered together with agronomic, phenotypic, and quality-related traits [20].

Volatile profiling provided an additional biochemical perspective by revealing variation in the relative abundances of six tentatively identified compounds among four representative cultivars. Although preliminary, these results demonstrate the potential of combining molecular marker analysis with volatile profiling to characterize genetic and biochemical diversity within rice germplasm. Further evaluation of larger germplasm collections using comprehensive metabolomic analyses and independent biological replication would help establish the consistency and breeding relevance of these patterns.

The ISSR fragment amplified using primer UBC818 (1250 bp) and the SRAP fragment amplified using primer combination Me4/Em5 (300 bp) showed potential associations with aromatic rice cultivars and represent candidate fragments for further marker development. Although both are dominant markers and have not been sequenced, sequence characterization and validation across diverse rice germplasm are necessary to determine whether these fragments are consistently associated with fragrance-related traits and assess their potential utility in cultivar characterization and marker-assisted selection. Their relationship with aroma-associated loci, including *BADH2*, remains to be established.

Overall, integrating molecular marker data with volatile profiling offers a complementary framework for characterizing Thai rice germplasm and identifying accessions of potential value for conservation and breeding. Although the present study does not establish a direct relationship between genetic variation and aroma-related traits, the combined approach provides a foundation for future investigations integrating high-resolution genomic data, phenotypic evaluation, and metabolomic profiling to support germplasm conservation, cultivar characterization, and the selection of breeding materials with desirable quality traits.

## 4. Materials and Methods

### 4.1. Rice Materials

Twenty-nine Thai rice cultivars, comprising 14 aromatic and 15 non-aromatic varieties, were selected for this study (Table 2). Seeds were obtained from the National Rice Seed Storage and Research Center, Rice Department, Thailand. The cultivars represented diverse Thai rice germplasm originating from different geographical regions and differing in aroma, pericarp pigmentation, and endosperm type. Seeds of each cultivar were germinated under controlled conditions at the Department of Biology, Faculty of Science, Mahasarakham University, Thailand. At 15 days after germination, young leaves were collected from five healthy seedlings of each cultivar. The leaves from the five seedlings were pooled to form a single composite sample per cultivar before genomic DNA extraction for subsequent multilocus DNA fingerprinting analyses.

For volatile compound analysis, four cultivars were selected based on the results of the molecular analyses to represent genetically distinct rice groups. Approximately 10 g of mature milled rice grains from each selected cultivar were used for HS-GC–MS analysis. Prior to analysis, the rice grains were stored in airtight containers under dry conditions at room temperature to minimize changes in volatile composition.

### 4.2. Genomic DNA Extraction, PCR Amplification of ISSR, SCoT and SRAP Markers

Genomic DNA was extracted from young rice leaves using a modified cetyltrimethylammonium bromide (CTAB) method based on Murray and Thompson (1980) [55], with minor modifications. Approximately 0.2 g of fresh leaf tissue from each accession was excised, immediately frozen in liquid nitrogen, and ground to a fine powder using a pre-chilled mortar and pestle. The powdered tissue was transferred to a 1.5 mL microcentrifuge tube containing 600 μL of preheated CTAB extraction buffer (65 °C), followed by the addition of 10 μL β-mercaptoethanol (Sigma-Aldrich Inc., St. Louis, MO, USA). Samples were gently mixed and incubated at 65 °C for 45 min, with gentle inversion every 5 min. Subsequently, 600 μL of chloroform:isoamyl alcohol (24:1, *v*/*v*) was added, and the mixtures were gently inverted before centrifugation at 12,000× *g* for 15 min. The aqueous phase was carefully transferred to a fresh tube, and genomic DNA was precipitated by adding cold isopropanol. After incubation at −20 °C for 30 min, the samples were centrifuged at 12,000× *g* for 10–15 min to recover the DNA pellet.

The DNA pellet was washed with 500 μL of cold 70% ethanol, centrifuged at 12,000× *g* for 10 min, air-dried at room temperature, and resuspended in 50 μL TE buffer. The suspension was incubated at 37 °C for 20 min, followed by treatment with 2 μL RNase A (Bio-Helix Co., Ltd., New Taipei City, Taiwan) and a further incubation at 37 °C for 30 min to remove residual RNA. DNA quality and integrity were assessed by electrophoresis on a 1.0% agarose gel prepared in 1× TBE buffer and stained with Prime Juice™ Preloading Fluorescent Stain (Bio-Helix Co., Ltd., New Taipei City, Taiwan). The gels were visualized using a GelDoc Go Gel Imaging System (Bio-Rad Laboratories, Inc., Hercules, CA, USA). DNA concentration and purity were measured with a NanoDrop Lite Plus Spectrophotometer (Thermo Fisher Scientific, Madison, WI, USA). All DNA samples were diluted to a working concentration of 50 ng μL^−1^ using nuclease-free water (Invitrogen™, Thermo Fisher Scientific, Carlsbad, CA, USA) and stored at −20 °C until use.

Genetic diversity was evaluated using ISSR, SCoT, and SRAP markers. PCR amplifications were carried out in a Biometra TAdvanced thermal cycler (Bio-Active Co., Ltd., Bangkok, Thailand). The primer sequences, PCR reaction mixtures, and thermal cycling conditions are presented in Table 5. Amplified PCR products were separated by electrophoresis on 1.5% agarose gels prepared in 1× TBE buffer and stained with Prime Juice™ (BIO-HELIX Co., LTD., New Taipei City, Taiwa). A 100 bp DNA ladder (BIO-HELIX Co., LTD., New Taipei City, Taiwan) was used as the molecular size marker. Electrophoresis was performed at 80 V for 90 min, and DNA bands were visualized using a GelDoc Go Gel Imaging System (Bio-Rad Laboratories, Inc., Hercules, CA, USA). A non-template control (NTC) was included in every PCR run to monitor contamination, and all PCR amplifications were performed in duplicate to verify reproducibility.

### 4.3. Molecular Marker Data Analysis

DNA fingerprinting profiles generated using ISSR, SCoT, and SRAP markers were independently scored for all 29 Thai rice cultivars, comprising aromatic and non-aromatic germplasm. The sizes of amplified DNA fragments were estimated by comparison with a 100 bp DNA ladder using PhotoCaptMW software (version 99.04; Vilber Lourmat, Marne-la-Vallée, France). Only clear and reproducible amplification products were scored, with each DNA fragment recorded in a binary matrix as present (1) or absent (0).

Genetic similarity among rice cultivars was estimated using Jaccard’s similarity coefficient [57]. Binary data matrices were generated separately for each marker system and then combined into an integrated dataset for comprehensive genetic diversity analysis. Cluster analysis was performed using the Unweighted Pair Group Method with Arithmetic Mean (UPGMA), based on the Sequential, Agglomerative, Hierarchical, and Nested (SAHN) clustering algorithm implemented in NTSYSpc version 2.1 [58].

The discriminatory efficiency of each marker system was evaluated by calculating the total number of bands (NTB), number of polymorphic bands (NPB), number of monomorphic bands (NMB), percentage of polymorphic bands (PP), polymorphic information content (PIC), resolving power (RP), and marker index (MI) according to previously described methods [28,59,60,61,62]. The percentage of polymorphism (PP) was calculated as the proportion of polymorphic bands relative to the total number of amplified bands. The polymorphic information content (PIC) of each primer was calculated using the formula PIC = 2*f_i_*(1 − *f_i_*), where *f_i_* is the frequency of accessions possessing a given band and (1 − *f_i_*) is the frequency of accessions lacking that band [59]. The resolving power (RP) of each primer was calculated as RP = Σ*I_β_*, where *I_β_* = 1 − 2|0.5 − *p*| and *p* represents the proportion of accessions in which the band was present [62]. The marker index (MI) was calculated as MI = PIC × NPB, where NPB is the number of polymorphic bands generated by each primer [58]. Expected heterozygosity (*H_e_*), also referred to as Nei’s gene diversity, was estimated as *H_e_* = 1−∑*pi*^2^, where *pi* represents the frequency of the *i^th^* allele [62].

### 4.4. Multivariate Analysis and Visualization of Molecular Marker Data

The combined binary dataset generated from ISSR, SCoT, and SRAP markers was used to investigate the genetic relationships among the 29 Thai rice cultivars. Amplified fragments were scored as present (1) or absent (0), and pairwise genetic similarities were calculated using Jaccard’s similarity coefficient. Hierarchical clustering was performed using the UPGMA to classify cultivars according to their genome-wide molecular marker profiles. A clustered heatmap was constructed to visualize the distribution of binary-scored marker loci among cultivars, with rows representing rice cultivars and columns representing marker loci. The dendrogram and heatmap facilitated the identification of genetic clustering patterns and the differentiation of aromatic and non-aromatic rice cultivars.

To further examine the genetic relationships among cultivars, principal coordinate analysis (PCoA) was performed using the same combined binary marker dataset. A genetic distance matrix based on Jaccard’s similarity coefficient was subjected to eigenvalue decomposition, and the first two principal coordinates (PCoA1 and PCoA2) were used to visualize the genetic relationships among cultivars in a two-dimensional space. The ordination plot was used to assess the genetic differentiation and clustering patterns between aromatic and non-aromatic rice cultivars identified by the molecular markers.

The discriminatory performance of the ISSR, SCoT, and SRAP marker systems was further compared using primer-based values of PIC, RP, and MI. The distributions of these parameters were visualized using boxplots, in which the central horizontal line represents the median, the box indicates the interquartile range (IQR), whiskers extend to 1.5 × IQR, and individual points correspond to each primer or primer combination. All statistical analyses and graphical visualizations, including hierarchical clustering, heatmap construction, principal coordinate analysis, and boxplot generation, were performed in R version 4.3.2 [61] using the pheatmap, vegan, and ggplot2 packages.

### 4.5. Untargeted Profiling of Volatile Compounds

Untargeted profiling of volatile organic compounds (VOCs) was performed using HS-GC–MS, a widely used approach for characterizing aroma-related volatile compounds in rice [38,48]. Approximately 10 g of milled rice grains from each selected cultivar were transferred into 20 mL headspace glass vials. Distilled water was added at a rice-to-water ratio of 1:2 (*w*/*v*). The vials were immediately sealed with polytetrafluoroethylene (PTFE)/silicone septa, and the rice was cooked directly in the sealed vials at 100 °C for 15 min. After cooking, the samples were allowed to cool at room temperature for 10 min. The vials remained sealed until analysis to minimize the loss or alteration of volatile compounds. Analyses were performed using an Agilent 8890 gas chromatograph coupled with an Agilent 5977C mass selective detector (Agilent Technologies, Santa Clara, CA, USA) and equipped with a PAL RTC 120 Series 2 headspace autosampler (CTC Analytics, Zwingen, Switzerland). Before injection, the sealed vials were incubated at 80 °C for 15 min with agitation at 250 rpm, using a cycle of 5 s of agitation followed by 2 s of rest. Subsequently, 2.5 mL of headspace gas was automatically aspirated from each vial and introduced directly into the GC–MS system. Volatile profiling was conducted as a preliminary comparative analysis of the selected rice cultivars. A single cooked-rice sample was analyzed for each cultivar, without independent biological replication. Accordingly, the results were interpreted descriptively to compare the observed volatile profiles rather than to assess biological variation or statistical significance among cultivars.

Chromatographic data were processed using Agilent MassHunter Qualitative Analysis software (version 12.0; Agilent Technologies). Six volatile compounds were tentatively identified by comparing their mass spectra with reference spectra in the Wiley23 and NIST23 mass spectral libraries. Library match scores ranged from 71.43% to 90.85%, with 4-methylbenzaldehyde showing the lowest score (71.43%). Retention indices were also compared with published reference values to provide complementary evidence supporting the annotations. The compounds were selected based on their library match scores and occurrence in the chromatographic profiles. Because authentic reference standards were unavailable and blank samples were not analyzed, the compound identities should be regarded as tentative. Relative GC–MS peak areas were used to characterize and compare the volatile profiles of the four analyzed samples. As authentic reference standards and external calibration curves were unavailable, absolute compound concentrations were not determined. The relative peak-area data provided a basis for descriptive comparisons and exploratory multivariate analyses, offering preliminary insights into variation in volatile composition among the analyzed samples.

### 4.6. Statistical Analysis of Volatile Compound Profiles

Relative peak areas obtained by HS-GC–MS were used to characterize the volatile profiles of the four selected rice cultivars. In the absence of authentic reference standards and external calibration curves, compound abundances were assessed semi-quantitatively, and absolute compound concentrations were not determined. Relative peak area (%) was calculated as 100 × the integrated peak area of a compound divided by the total integrated chromatographic peak area for that sample. The six reported percentages were not renormalized to sum to 100%. The relative abundances (%) of the six tentatively identified volatile compounds were used for descriptive comparisons and exploratory multivariate analyses.

The PCA input matrix comprised four analyzed samples as rows and six compounds as columns, using relative peak areas (%) from Table 4. PCA was performed in R using the prcomp() function with scale. = TRUE, which applies variable-wise (compound-wise) standardization across cultivars before calculating the principal components. HCA was performed on the compound-wise standardized relative peak areas (%) using Euclidean distance and Ward’s minimum variance method (Ward.D2). For heatmap visualization using the pheatmap package, the data matrix was arranged with volatile compounds as rows and cultivars as columns, and scale = "row" was applied to standardize each compound across the four cultivars. Thus, both analyses used compound-wise standardization to facilitate comparisons of relative abundance patterns among the analyzed samples. In the heatmap, red and blue indicate positive and negative standardized values, respectively, representing relative peak areas above and below the mean for each compound across the four cultivars. Volatile compounds were manually classified according to their predominant chemical classes for biological interpretation; these classifications were used solely for annotation and were not included as variables in the clustering analysis.

Volatile profiling was performed once for each cultivar. Accordingly, PCA and HCA were based on four cultivar-level observations and six tentatively identified volatile compounds. The resulting patterns were interpreted as exploratory descriptions of the observed volatile profiles rather than as evidence of statistically supported differences among cultivars. All statistical analyses and graphical visualizations were performed using R version 4.2.2 [63].

## 5. Conclusions

This study integrated ISSR, SCoT, and SRAP molecular markers with HS-GC–MS-based volatile profiling to characterize genetic and biochemical variation among Thai rice cultivars. SRAP showed the highest values for several marker informativeness parameters among the three marker systems, while the combined marker dataset provided complementary information for characterizing genetic relationships and distinguishing major groups among aromatic and non-aromatic cultivars. Volatile profiling of four representative cultivars further revealed differences in the relative abundances of aldehydes, a carboxylic acid, and an ester among the analyzed samples. Hexanal was the predominant volatile compound across all four cultivars, while the analyzed KDML105 sample showed a distinct volatile profile, with relatively higher abundances of acetic acid, 4-methylbenzaldehyde, and methyl salicylate. These findings highlight the complementary value of integrating multilocus molecular markers and volatile profiling to characterize the genetic and biochemical diversity of Thai rice germplasm. This integrated approach supports germplasm characterization and the selection of genetically diverse cultivars for further investigation of aroma-related traits. The exploratory volatile profiles obtained from one cooked-rice sample per cultivar add a biochemical dimension to the molecular characterization and provide a foundation for further evaluation of aroma-related variation using independently replicated samples. Although volatile profiling was limited to four cultivars, the observed patterns provide preliminary insights into variation in volatile composition and a foundation for further exploring potential relationships between genetic diversity and volatile profiles. Furthermore, 2AP was not detected under the analytical conditions used, highlighting the importance of targeted analysis to further characterize this key rice aroma compound. Future studies involving larger and more diverse germplasm collections, high-density genomic analyses, independent biological replication, quantitative determination of key aroma compounds, and comprehensive volatile metabolomic profiling will help validate and extend these findings, deepen understanding of the genetic and biochemical basis of aroma variation, and strengthen the application of molecular and biochemical characterization in rice germplasm management and quality-oriented breeding.

## Figures and Tables

**Table 1:** Characteristics and genetic diversity parameters of ISSR, SCoT, and SRAP markers used for the analysis of 29 Thai rice cultivars.

Marker	Primer	NTB	NPB	NMB	Product Size (bp)	PP	PIC	RP	MI	*H_e_*	Specific Band
ISSR	UBC811	5	1	4	200–1300	20.00	0.067	0.620	0.340	0.086	-
	UBC817	7	2	5	300–1300	28.57	0.127	1.450	0.890	0.124	-
	UBC818	7	4	3	400–1250	57.14	0.137	1.930	0.960	0.172	1250 bp
	UBC827	5	3	2	500–1200	60.00	0.121	1.240	0.610	0.150	-
	UBC834	6	4	2	200–1100	66.67	0.208	2.280	1.250	0.262	-
	UBC835	5	2	3	450–1400	40.00	0.132	1.240	0.660	0.167	-
	UBC840	5	2	3	200–1400	33.33	0.141	1.450	0.840	0.183	-
	UBC848	4	1	3	300–1600	25.00	0.087	0.690	0.350	0.113	-
	UBC859	5	0	5	220–680	0.00	0.000	0.000	0.000	0.000	-
	UBC888	5	1	4	420–1300	20.00	0.070	0.690	0.350	0.090	-
	Total	54	20	35		350.71	1.090	11.586	6.240	1.346	
	Mean	5.4	2.00	3.50		35.07	0.109	1.159	0.624	0.135	
SCoT	SCoT6	6	2	4	200–1300	33.33	0.119	1.590	0.710	0.155	-
	SCoT8	5	2	3	250–1600	40.00	0.106	0.830	0.530	0.127	-
	SCoT15	6	3	3	100–200	50.00	0.151	1.790	0.900	0.189	-
	SCoT17	7	5	2	180–1300	71.43	0.193	2.280	1.350	0.238	-
	SCoT18	4	2	2	220–650	50.00	0.162	1.310	0.650	0.206	-
	SCoT21	5	3	2	400–1600	60.00	0.061	0.340	0.300	0.065	-
	SCoT22	5	2	3	180–1600	40.00	0.142	1.520	0.710	0.185	-
	SCoT27	5	4	1	200–1000	80.00	0.197	1.520	0.980	0.236	-
	SCoT29	6	2	4	200–1000	33.33	0.116	1.380	0.700	0.150	-
	SCoT30	5	3	2	200–650	60.00	0.174	1.520	0.870	0.215	-
	Total	54	28	26		518.10	1.419	14.069	7.707	1.765	
	Mean	5.40	2.80	2.60		51.81	0.142	1.407	0.771	0.176	
SRAP	Me1/Em1	6	6	0	250–1400	100.00	0.339	4.000	2.040	0.434	-
	Me1/Em2	5	2	3	500–1600	40.00	0.076	0.480	0.380	0.085	-
	Me2/Em2	7	6	1	200–1100	85.71	0.217	2.480	1.520	0.264	-
	Me2/Em3	5	4	1	180–1500	80.00	0.232	2.140	1.160	0.289	-
	Me3/Em3	6	5	1	100–1700	83.33	0.259	2.690	1.550	0.323	-
	Me3/Em4	5	3	2	100–1400	60.00	0.109	0.970	0.540	0.133	-
	Me4/Em4	7	6	1	100–900	85.71	0.197	2.140	1.380	0.236	-
	Me4/Em5	7	5	2	200–1300	71.43	0.153	1.930	1.070	0.189	300 bp
	Me5/Em5	7	6	1	100–800	85.71	0.277	3.860	1.940	0.355	-
	Me5/Em1	7	7	0	150–1400	100.00	0.285	4.000	1.990	0.361	-
	Total	62	50	12		791.89	2.143	24.690	13.570	2.668	
	Mean	6.20	5.00	1.20		79.19	0.214	2.469	1.357	0.267	

Note: NTB, total number of bands; NPB, number of polymorphic bands; NMB, number of monomorphic bands; PP, percentage of polymorphic bands; PIC, polymorphic information content; RP, resolving power; MI, marker index; *H_e_*, expected heterozygosity. Aroma-associated bands refer to amplification fragments consistently detected in all aromatic rice cultivars examined but absent from all non-aromatic cultivars.

**Table 3:** Pairwise Jaccard genetic similarity coefficients among 29 Thai rice cultivars based on the combined ISSR, SCoT, and SRAP marker dataset.

	C1	C2	C3	C4	C5	C6	C7	C8	C9	C10	C11	C12	C13	C14	C15	C16	C17	C18	C19	C20	C21	C22	C23	C24	C25	C26	C27	C28	C29
C1	1.00																												
C2	0.82	1.00																											
C3	0.88	0.87	1.00																										
C4	0.80	0.85	0.84	1.00																									
C5	0.81	0.83	0.81	0.81	1.00																								
C6	0.80	0.82	0.81	0.85	0.79	1.00																							
C7	0.83	0.82	0.83	0.78	0.88	0.77	1.00																						
C8	0.83	0.81	0.91	0.82	0.79	0.81	0.84	1.00																					
C9	0.79	0.82	0.83	0.85	0.83	0.83	0.82	0.85	1.00																				
C10	0.69	0.72	0.72	0.74	0.64	0.73	0.65	0.69	0.69	1.00																			
C11	0.83	0.85	0.83	0.81	0.83	0.78	0.81	0.81	0.82	0.70	1.00																		
C12	0.83	0.81	0.87	0.84	0.80	0.84	0.81	0.89	0.90	0.70	0.82	1.00																	
C13	0.66	0.74	0.73	0.77	0.69	0.73	0.69	0.68	0.74	0.86	0.70	0.72	1.00																
C14	0.68	0.71	0.72	0.75	0.65	0.72	0.64	0.65	0.67	0.82	0.69	0.69	0.83	1.00															
C15	0.74	0.67	0.74	0.66	0.68	0.69	0.71	0.72	0.70	0.60	0.70	0.73	0.58	0.60	1.00														
C16	0.74	0.66	0.71	0.63	0.67	0.67	0.68	0.68	0.66	0.60	0.69	0.70	0.55	0.59	0.90	1.00													
C17	0.68	0.64	0.70	0.66	0.63	0.65	0.64	0.68	0.65	0.70	0.65	0.68	0.69	0.71	0.72	0.71	1.00												
C18	0.59	0.58	0.62	0.62	**0.54**	0.60	0.56	0.58	0.59	0.67	0.58	0.63	0.66	0.71	0.74	0.74	0.72	1.00											
C19	0.74	0.63	0.71	0.66	0.63	0.69	0.67	0.72	0.67	0.63	0.66	0.72	0.60	0.60	0.81	0.80	0.76	0.69	1.00										
C20	0.64	0.59	0.62	0.66	0.59	0.65	0.59	0.63	0.60	0.74	0.59	0.67	0.71	0.73	0.70	0.70	0.76	0.81	0.71	1.00									
C21	0.62	0.60	0.64	0.66	0.59	0.65	0.58	0.62	0.60	0.73	0.60	0.65	0.71	0.73	0.68	0.71	0.79	0.80	0.71	0.90	1.00								
C22	0.70	0.65	0.69	0.69	0.68	0.69	0.68	0.68	0.66	0.63	0.70	0.70	0.60	0.63	0.81	0.81	0.76	0.70	0.76	0.71	0.73	1.00							
C23	0.77	0.67	0.72	0.68	0.67	0.68	0.68	0.72	0.68	0.59	0.72	0.72	0.57	0.61	0.89	0.84	0.74	0.72	0.83	0.68	0.67	0.83	1.00						
C24	0.74	0.69	0.76	0.69	0.68	0.69	0.69	0.72	0.68	0.60	0.71	0.72	0.58	0.63	0.91	0.90	0.73	0.73	0.81	0.69	0.70	0.82	0.90	1.00					
C25	0.70	0.63	0.72	0.66	0.64	0.68	0.66	0.74	0.69	0.58	0.66	0.73	0.57	0.59	0.88	0.84	0.75	0.73	0.86	0.70	0.73	0.81	0.87	0.89	1.00				
C26	0.72	0.66	0.71	0.67	0.67	0.66	0.65	0.72	0.68	0.56	0.69	0.72	0.56	0.57	0.87	0.84	0.75	0.71	0.82	0.68	0.68	0.80	**0.92**	0.88	0.88	1.00			
C27	0.71	0.66	0.72	0.66	0.64	0.66	0.65	0.73	0.67	0.58	0.68	0.72	0.56	0.60	0.89	0.89	0.74	0.74	0.81	0.70	0.71	0.81	0.89	0.91	0.91	0.91	1.00		
C28	0.65	0.57	0.64	0.61	0.56	0.60	0.59	0.65	0.59	0.61	0.61	0.63	0.59	0.64	0.79	0.78	0.73	0.76	0.76	0.76	0.75	0.74	0.81	0.80	0.80	0.81	0.85	1.00	
C29	0.67	0.62	0.66	0.63	0.60	0.60	0.60	0.66	0.63	0.64	0.63	0.66	0.63	0.68	0.79	0.79	0.74	0.76	0.73	0.74	0.72	0.74	0.81	0.81	0.78	0.83	0.86	0.90	1.00

Note: The lowest and highest Jaccard genetic similarity coefficients are indicated in bold. C1: RD6; C2: RD15; C3: RD18; C4: RD33; C5: RD45; C6: RD77; C7: RD83; C8: RD97; C9: KDML105; C10: Khao Jao Hom Suphanburi; C11: Khao Niao RD6; C12: PTT1; C13: Khao Mali Nin Surin; C14: Khao Hom Bai Toey 62; C15: RD31; C16: RD41; C17: RD43; C18: RD53; C19: RD57; C20: RD61; C21: RD63; C22: RD71; C23: RD79; C24: RD81; C25: RD85; C26: RD87; C27: RD91; C28: RD95; and C29: RD101. Jaccard similarity coefficients and UPGMA clustering were calculated using the vegan package in R version 4.3.2.

**Table 4:** Putatively annotated volatile compounds detected in cooked rice cultivars (KDML105, RD43, RD79, and PTT1) by static headspace gas chromatography–mass spectrometry (HS-GC–MS) based on Agilent MassHunter library matching.

ID	Compound	Formula	CAS	Class	RT (min)	%Library Match	KDML 105 area	% Peak Area	RD43 Area	%RD43 Area	RD79 Area	%RD79 Area	PTT1 Area	%PTT1 Area	Aroma Descriptor	Importance in Rice Aroma	Reference
1	Hexanal	C_6_H_12_O	66-25-1	Aldehyde	9.35–9.36	86.25	487,671	19.51	1,539,815	40.79	785,904	51.30	1,311,954	49.64	Green, grassy, fresh	Major lipid-derived aldehyde contributing green and grassy notes to cooked rice aroma.	[38,47]
2	Heptanal	C_7_H_14_O	111-71-7	Aldehyde	24.99–25.02	87.87	22,229	0.89	54,475	1.44	30,879	2.02	34,815	1.32	Green, fatty, citrus	Minor lipid-derived aldehyde contributing green and fatty notes to cooked rice aroma.	[38,47]
3	Acetic acid	C_2_H_4_O_2_	64-19-7	Carboxylic acid	30.55–30.82	90.85	80,375	3.22	43,016	1.14	30,150	1.97	44,651	1.69	Sour, acidic, vinegar-like	Contributes acidic and fermented notes to cooked rice aroma.	[48]
4	Nonanal	C_9_H_18_O	124-19-6	Aldehyde	33.65–33.73	89.30	44,221	1.77	50,164	1.33	28,616	1.87	38,415	1.45	Fatty, citrus, floral	Lipid-derived aldehyde contributing fatty, citrus and floral notes to cooked rice aroma.	[47,49]
5	4-Methylbenzaldehyde	C_8_H_8_O	104-87-0	Aromatic aldehyde	49.63–49.65	71.43	61,744	2.47	44,945	1.19	14,951	0.98	42,011	1.59	Sweet, almond like, floral	Minor aromatic aldehyde contributing sweet and floral notes.	[38,49]
6	Methyl salicylate	C_8_H_8_O_3_	119-36-8	Ester	52.33–52.91	81.79	88,831	3.55	43,674	1.16	0	0.00	52,714	1.99	Minty, sweet, wintergreen	Minor ester contributing sweet, minty and floral notes to cooked rice aroma.	[38,50]

Note: Volatile compounds were tentatively identified by HS-GC–MS using Agilent MassHunter Qualitative Analysis software through comparison of their mass spectra with reference spectra in the NIST and Wiley mass spectral libraries. Library match (%) represents the spectral similarity between the experimental and reference spectra. Retention times (RT) are presented as the minimum–maximum values observed among the four rice cultivars. Peak areas and relative abundances (%) were obtained directly from chromatographic integration using Agilent MassHunter software.

**Table 5:** Primer sequences, PCR reaction mixtures, and thermal cycling conditions for ISSR, SCoT, and SRAP markers used to assess the genetic diversity of 29 Thai rice cultivars.

	Name	Primer Sequence (5′-3′)	Reaction Mixture (25 µL Total Volume)	PCR Protocol	Reference
ISSR	UBC811	5′-GAGAGAGAGAGAGAGAC-3′	The PCR mixture contained 1.2X OmniPCR Supermix 15 ul, 50 pmole/ul of primer 1.5 ul, and 50–100 ng genomic DNA 1 ul, with the final volume adjusted using nuclease-free water	Pre-denaturation (1 cycle) 94 °C for 5 min Denaturation, Annealing, Extension (35 cycles) 94 °C for 45 s 53 °C for 45 s 72 °C for 1.5 min Final Extension (1 cycle) 72 °C for 10 min	[27]
	UBC817	5′-CACACACACACACACAA-3′			
	UBC818	5′-CACACACACACACACAC-3′			
	UBC827	5′-ACACACACACACACACG-3′			
	UBC834	5′-AGAGAGAGAGAGAGAGYT-3′			
	UBC835	5′-AGAGAGAGAGAGAGAGYC-3′			
	UBC840	5′-GAGAGAGAGAGAGAGAYT-3′			
	UBC848	5′-CACACACACACACACARG-3′			
	UBC859	5′-TGTGTGTGTGTGTGTGRC-3′			
	UBC888	5′-BDBCACACACACACACA-3′			
SCoT	SCoT6	5′-CAACAATGGCTACCACGC-3′	The PCR mixture contained 1.2X OmniPCR Supermix 15 ul, 50 pmole/ul of primer 1.5 ul, and 50–100 ng genomic DNA 1 ul, with the final volume adjusted using nuclease-free water	Pre-denaturation (1 cycle) 95 °C for 5 min Denaturation, Annealing, Extension (35 cycles) 95 °C for 45 s 57 °C for 45 s 72 °C for 1.5 min Final Extension (1 cycle) 72 °C for 10 min	[56]
	SCoT8	5′-CAACAATGGCTACCACGT-3′			
	SCoT15	5′-ACGACATGGCGACCGCGA-3′			
	SCoT17	5′-ACCATGGCTACCACCGAG-3′			
	SCoT18	5′-ACCATGGCTACCACCGCC-3′			
	SCoT21	5′-ACGACATGGCGACCCACA-3′			
	SCoT22	5′-AACCATGGCTACCACCAC-3′			
	SCoT27	5′-ACCATGGCTACCACCGTG-3′			
	SCoT29	5′-CCATGGCTACCACCGGCC-3′			
	SCoT30	5′-CCATGGCTACCACCGGCG-3′			
SRAP	Me1/Em1	Me1-F 5′-TGAGTCCAAACCGGATA-3′	The PCR mixture contained 1X OmniPCR Supermix 15 ul, 50 pmole/ul each forward and reward primer 1.5 ul, and 50–100 ng genomic DNA 1 ul, with the final volume adjusted using nuclease-free water	Pre-denaturation (1 cycle) 94 °C for 5 min Denaturation, Annealing, Extension (35 cycles) 95 °C for 45 s 54 °C for 45 s 72 °C for 1.5 min Final Extension (1 cycle) 72 °C for 10 min	[28]
		Em1-R 5′-GACTGCGTACGAATTTGC-3′			
	Me1/Em2	Me1-F 5′-TGAGTCCAAACCGGATA-3′			
		Em2-R 5′-GACTGCGTACGAATTGCA-3′			
	Me2/Em2	Me2-F 5′-TGAGTCCAAACCGGAGC-3′			
		Em2-R 5′-GACTGCGTACGAATTGCA-3′			
	Me2/Em3	Me2-F 5′-TGAGTCCAAACCGGAGC-3′			
		Em3-R 5′-GACTGCGTACGAATTAGC-3′			
	Me3/Em3	Me3-F 5′-TGAGTCCAAACCGGACC-3′			
		Em3-R 5′-GACTGCGTACGAATTAGC-3′			
	Me3/Em4	Me3-F 5′-TGAGTCCAAACCGGACC-3′			
		Em4-R 5′-GACTGCGTACGAATTTAG-3′			
	Me4/Em4	Me4-F 5′-TGAGTCCAAACCGGTAG-3′			
		Em4-R 5′-GACTGCGTACGAATTTAG-3′			
	Me4/Em5	Me4-F 5′-TGAGTCCAAACCGGTAG-3′			
		Em5-R 5′-GACTGCGTACGAATTGGT-3′			
	Me5/Em5	Me5-F 5′-TGAGTCCAAACCGGTGT-3′			
		Em5-R 5′-GACTGCGTACGAATTGGT-3′			
	Me5/Em1	Me5-F 5′-TGAGTCCAAACCGGTGT-3′			
		Em1-R 5′-GACTGCGTACGAATTTGC-3′			

## Data Availability

The original contributions presented in this study are included in the article. Further inquiries can be directed to the corresponding authors.

## Supplementary Materials

The following supporting information can be downloaded at: https://www.mdpi.com/article/10.3390/plants15213482/s1, Table S1: Binary marker matrix (0/1) for 29 Thai rice cultivars based on ISSR, SCoT, and SRAP markers. Table S2: Pairwise Jaccard genetic similarity coefficients among the 29 cultivars based on the combined marker dataset. Figure S1: Principal coordinate analysis (PCoA) plot showing genetic relationships among the 29 Thai rice cultivars, with cultivar labels.
